# The stress response factor SigH mediates intrinsic resistance to multiple antibiotics in *Mycobacterium abscessus*

**DOI:** 10.1128/spectrum.03965-25

**Published:** 2026-06-15

**Authors:** Md Shah Alam, Mst Sumaia Khatun, Buhari Yusuf, Lijie Li, Belachew Aweke Mulu, Haftay Abraha Tadesse, Jingran Zhang, Xirong Tian, Cuiting Fang, Yamin Gao, Zhiyong Liu, Jinxing Hu, Xinwen Chen, Nanshan Zhong, Liqiang Feng, H. M. Adnan Hameed, Shuai Wang, Tianyu Zhang

**Affiliations:** 1 State Key Laboratory of Respiratory Disease, Guangzhou Institutes of Biomedicine and Health, Chinese Academy of Sciences 74627, Guangzhou, China; 2 University of Chinese Academy of Sciences 74519, Beijing, China; 3 Guangdong-Hong Kong-Macao Joint Laboratory of Respiratory Infectious Diseases, Guangzhou Institutes of Biomedicine and Health, Chinese Academy of Sciences 74627, Guangzhou, China; 4 China-New Zealand Joint Laboratory on Biomedicine and Health, Guangzhou, China; 5 School of Life Sciences, University of Science and Technology of China 12652, Hefei, China; 6 State Key Laboratory of Respiratory Disease, National Clinical Research Center for Respiratory Disease, The National Center for Respiratory Medicine, Guangzhou Medical University 26468, Guangzhou, China; 7 State Key Laboratory of Respiratory Disease, Guangzhou Chest Hospital, Guangzhou, China; 8 Guangzhou National Laboratory 612039, Guangzhou, China; University of Guelph College of Biological Science, Guelph, Canada

**Keywords:** non-tuberculous mycobacteria, *M. abscessus*, SigH, intrinsic resistance, gene expression regulation

## Abstract

*Mycobacterium abscessus* (Mab) is a pathogenic fast-growing mycobacterium with complex, poorly understood drug resistance mechanisms and limited treatment options. This warrants renewed efforts to dissect resistance mechanisms and identify novel targets. Here, we report that disruption of the putative sigma factor *sigH* in Mab results in hypersensitivity to levofloxacin, moxifloxacin, tigecycline, tetracycline, amikacin, vancomycin, and rifabutin. Mab SigH shares 84% peptide sequence identity with *Mycobacterium tuberculosis* (Mtb) SigH, a well-known stress-response protein and global transcriptional regulator. Complementation of the knockout strain (Δ*sigH*) with Mab *sigH* or its Mtb homolog restored the drug resistance phenotype. Additionally, Δ*sigH* showed increased sensitivity to oxidative and heat stress compared to the wild-type (WT) Mab and complemented strains. Transcriptomic analysis revealed that the deletion of *sigH* disrupted the balance of gene expression, primarily elevating the expression of genes encoding YrbE and MCE family proteins and downregulating genes encoding ABC-type transporters, sigma and anti-sigma factors, and other genes associated with antimicrobial resistance. Notably, Δ*sigH* accumulated more ethidium bromide and displayed cell-elongation phenotypes, both partially reversed by complementation. In addition, a native *sigH* promoter driving green fluorescent protein (GFP) produced 10-fold higher fluorescence in WT than in Δ*sigH*, suggesting that the presence of SigH drives higher GFP expression . Collectively, our findings identify SigH as a key regulator of global gene expression that links stress adaptation, cell-envelope homeostasis, and intrinsic multidrug resistance in Mab, hence representing a promising target for the development of novel therapeutics against Mab infections.

**IMPORTANCE:**

*Mycobacterium abscessus* (Mab) causes severe acute and chronic lung infections. The intrinsic drug resistance mechanisms of Mab remain poorly understood, posing significant therapeutic challenges. Here, we examined the role of the putative sigma factor SigH in intrinsic multidrug resistance in Mab. Mab SigH shares 84% amino acid identity with *M. tuberculosis*. SigH is a well-known stress response regulator. Transcriptomic analysis revealed that the deletion of *sigH* disrupted the balance of gene expression, primarily elevating the expression of genes encoding YrbE and MCE family proteins and downregulating genes encoding ABC-type transporters, sigma and anti-sigma factors, and other genes associated with antimicrobial resistance. A native *sigH* promoter driving green fluorescent protein (GFP) produced 10-fold higher fluorescence in wild type than in Δ*sigH*, suggesting that the presence of SigH drives higher GFP expression. Thus, SigH emerges as a central regulator integrating stress adaptation, envelope homeostasis, and multidrug resistance, offering a promising target for novel anti-Mab therapies.

## INTRODUCTION

*Mycobacterium abscessus* complex (MABC) comprises well-known, rapidly growing non-tuberculous mycobacteria (NTM) that can cause severe acute and chronic lung infections, particularly in patients with underlying lung diseases such as cystic fibrosis and obstructive pulmonary diseases, as well as skin and soft tissue infections (1, 2). Recently, MABC has been associated with a wide range of clinical manifestations due to increasing global morbidity and mortality rates (3). The intrinsic drug resistance mechanisms of MABC remain poorly understood, and the members of this complex, particularly *Mycobacterium abscessus* (Mab), pose a significant challenge in its infection therapy. To improve treatment options and combat prevalent Mab infections, novel drugs with new mechanisms of action are urgently needed.

MABC accounts for 2.6%–13.0% of all NTM-related pulmonary infections (4). Mab demonstrates intrinsic resistance to most therapeutic agents, and cure rates for Mab lung infections are very low (approximately 25%–58%), earning it the moniker “antibiotic nightmare” (5). Intrinsic drug resistance in Mab primarily stems from its thick, lipid-rich, and highly impermeable cell wall, active efflux pump systems, and enzymatic drug modification or inactivation mechanisms (6–8). For instance, aminoglycoside phosphotransferases and 2′-N-acetyltransferases catalyze the transfer of phosphate or acetyl groups to specific sites on aminoglycoside molecules, thereby neutralizing their antibacterial activity (9). Similarly, erythromycin resistance methylase mediates macrolide resistance, while MmpL5/MmpS5 confers resistance to bedaquiline and clofazimine (10). The MabTetX, a WhiB7-independent tetracycline-inactivating monooxygenase, increases Mab’s resistance to the tetracycline family (11). Additionally, spontaneous mutations in particular genes in response to drugs cause acquired resistance. For example, genetic polymorphism confers resistance to antibiotics like fluoroquinolones (FQs), which are broad-spectrum secondary therapeutic agents for multidrug-resistant tuberculosis and act by inhibiting DNA gyrase supercoiling activity (12, 13). Mab resistance to FQs is due to genetic alterations, especially in quinolone resistance-determining regions within DNA gyrase subunits GyrA and GyrB, the primary targets of FQs (14). Tigecycline (TIG) resistance in Mab has been linked to both SigH and RshA (15). Dysregulated environmental stress response sigma factor (SigH) is associated with tigecycline resistance in both *Mycobacterium tuberculosis* (Mtb) and Mab (16, 17). The limited success in anti-Mab drug discovery primarily stems from high intrinsic resistance and rapidly acquired resistance to currently available active drugs. However, the mechanisms underlying intrinsic drug resistance in Mab remain not fully elucidated (18).

WhiB7 is a redox-sensitive master transcriptional regulator crucial for activating intrinsic drug resistance systems in mycobacteria and other bacteria, such as *Streptomyces lividans* and *Rhodococcus jostii* (19, 20). The multi-drug resistance WhiB7 transcriptional regulator induces SigH transcription. SigH is an alternative sigma factor involved in the transcriptional regulation of genes responsible for mycobacterial stress responses, including oxidative, heat, and nitrosative stresses (21–24), and is negatively regulated by RshA (15, 25). The roles of SigH in stress adaptation and pathogenicity have been extensively studied in Mtb and *Mycobacterium smegmatis* (Msm) (23, 26), but remain largely unexplored in Mab.

In this study, we investigated the role of SigH (MAB_3543c) in antimicrobial resistance, stress response, and differential gene expression in Mab. Our findings provide new insights into the molecular mechanisms of intrinsic drug resistance and highlight SigH as a potential target for the development of novel therapeutic strategies against Mab infections.

## MATERIALS AND METHODS

### Bacterial strains and growth conditions

The Mab GZ002 (accession number CP034181), exhibiting a smooth phenotype (27), was cultivated at 37°C in a shaking incubator at 220 rpm. Growth media included Middlebrook 7H9 (Difco), supplemented with 0.2% glycerol, 0.05% Tween 80, and 10% OADC, or solid 7H10/7H11 agar supplemented with 0.5% glycerol and 10% OADC. *Escherichia coli* strain DH5α was propagated on solid or in liquid Luria-Bertani medium under identical conditions of 37°C and 220 rpm shaking, with an incubation period of 10–12 hours, while Mab GZ002 was grown on agar medium for 3–5 days. Antibiotic usage and their respective concentrations were tailored to the specific experimental demands.

### CRISPR-Cpf1-assisted knockout of *sigH* (*MAB_3543c*)

CRISPR-Cpf1-assisted recombineering was employed to knockout the Mab *sigH* gene via homology-directed repair (28–30). Specifically, the CRISPR RNA (*crsigH*) was designed as two complementary oligonucleotides, each 24 base pairs in length (*crsigH*-F/R), targeting a region adjacent to a protospacer adjacent motif (PAM) characterized by the 5′-YTN-3′ trinucleotide sequence. One oligonucleotide was designed with a *Hin*dIII overhang, while the other had a *Bpm*I overhang. These oligonucleotides were annealed and cloned into *Hin*dIII/*Bpm*I-digested pCRZEO (28) to generate pCRZEO-*crsigH*.

The homology-directed repair template (*sigH*UD) was constructed by amplifying 640-bp upstream (U) and 609-bp downstream (D) regions of the target gene, including flanking sequences from adjacent genes (Fig. S1). Both fragments were cloned into pBlueSK to generate pBlueSK-*sigH*UD. *sigH*UD was amplified from pBlueSK-*sigH*UD using the primer pair *sigH*UD-F/R (Table S1). pCRZEO-cr*sigH* and *sigH*UD were transformed into electrocompetent Mab cells harboring pJV53-Cpf1 (28) by electroporation. The transformants were then plated on a 7H11 plate containing zeocin (ZEO, 30 µg/mL), kanamycin (KAN, 100 µg/mL), and anhydrotetracycline (ATc, 100 ng/mL), and incubated at 30°C for 5 days. *sigH* knockout was verified by PCR and sequencing using the primer pair Id3543c-F/R (Fig. S1). To construct the selectable marker-free knockout strain, the knockout strain was grown in 7H9 medium and plated on a drug-free 7H10 plate to obtain single colonies. A colony that grew on drug-free plates but not on KAN- and ZEO-containing plates was selected as the unmarked strain (Δ*sigH*) to be used in downstream experiments (Fig. S2).

### Complementation and overexpression of *sigH*

Complemented (CP) strains were constructed by integrating *sigH* into the genome of Δ*sigH* to express the gene under its native promoter (*Np*) or *hsp60* promoter, or through ectopic expression—that is, cloning *sigH* or its Mtb homolog into pMV261 and transforming them into Δ*sigH*. For overexpression, pMV261-*sigH* was transformed into wild-type (WT) Mab. The transformants were grown on Middlebrook 7H10 agar plates supplemented with KAN at 100 µg/mL for 3–5 days at 37°C and were verified by PCR and sequencing. Thus, the following strains were used in this study: OEMab*sigH* (WT overexpressing Mab *sigH*), Δ*sigH* (selectable marker-free *sigH* knockout strain), CPMab*sigH* (the CP strain ectopically expressing Mab *sigH*), CPMtb*sigH* (the CP strain ectopically expressing Mtb *sigH*), CP*Np*Mab*sigH* (the CP strain in which *Np*-Mab *sigH* is integrated into Δ*sigH* genome), and CP*hsp60*Mab*sigH* (the CP strain in which *hsp60*-Mab *sigH* is integrated into Δ*sigH* genome). Results of Mab strain verification and all primer sequences are provided in the supplementary material (Fig. S3 and S4; Table S1).

### Drug susceptibility testing

Mab strains were routinely cultivated on Middlebrook 7H10 agar or in Middlebrook 7H9 broth. Antimicrobial agents, including TIG, tetracycline (TET), clarithromycin (CLA), clofazimine (CLF), vancomycin (VAN), amikacin (AMK), levofloxacin (LFX), moxifloxacin (MFX), imipenem (IMP), cefoxitin (CFX), rifabutin (RIB), and linezolid (LZD), were prepared as stock solutions and stored at −20°C. Broth microdilution assay, adhering to Clinical Laboratory Standards Institute (CLSI) guidelines, was employed to determine drug susceptibility (31, 32). Mycobacterial cultures were standardized to approximately 1 × 10^7^ colony-forming units per milliliter (CFU/mL) in 7H9 medium without Tween 80. Bacterial suspensions were subjected to two-fold serial dilutions in 96-well plates in the presence of individual drugs. These plates were incubated at 37°C for 3 days, with an extended incubation period of 14 days specifically for CLA, before assessing the endpoint. Minimum inhibitory concentrations (MICs) were determined according to CLSI criteria, defined as the minimal drug concentrations that visually inhibit mycobacterial growth. Additionally, spot culture agar methodology was also utilized for drug susceptibility assessments (33). Herein, WT, Δ*sigH*, and CPMab*sigH* strains were propagated in 7H9 medium at 37°C until an optical density (OD_600_ nm) of 0.6 was reached. Subsequently, 10-fold serial dilutions were applied and aliquoted onto plain Middlebrook 7H10 agar (drug-free serving as a control) alongside plates supplemented with varying drug concentrations. Following a 3-day incubation at 37°C, the plates were examined.

### Thiol-specific oxidative and heat stress assay

WT, Δ*sigH*, and CPMab*sigH* were grown to the exponential phase and equilibrated at an OD_600_ nm of 0.6 for oxidative and heat stress assays. For the oxidative stress assay, a 100 µL bacterial inoculum at OD_600_ nm 0.6 was spread on agar plates. Whatman paper disks (4.5 mm) were loaded with 10 µL of the oxidizing agent diamide at concentrations of 4 M, 2 M, 1 M, and 0.5 M. These disks were then placed on agar plates and incubated for 3 days at 37°C. The susceptibility of bacteria to diamide was assessed by measuring the zone of inhibition. Different survival curves were observed under heat and oxidative stress conditions. Survival under heat stress was determined based on CFU counts. Bacterial cultures were incubated in a water bath at 45°C, and at 1-hour intervals, 100 µL of the bacterial culture was diluted in PBS and plated on 7H10 agar plates to determine the viable cell number (34). Additionally, bacterial survival was assessed in the presence of H_2_O_2_ and diamide. The bacterial OD_600_ nm was adjusted to 0.5–0.6 and cultured with 50 mM diamide and 50 mM H_2_O_2_ individually, followed by incubation at 37°C. At 1-hour intervals, bacteria were 10-fold subjected to serial dilutions and plated to count CFU to determine the survival rates under stress conditions (23, 35).

### RNA preparation, sequencing, and transcriptomic analysis

RNA isolation and library preparation for RNA sequencing were performed on the WT, Δ*sigH*, and CPMab*sigH* strains. These bacterial strains were cultured in Middlebrook 7H9 medium supplemented with Tween 80 and incubated at 37°C until reaching the exponential growth phase, characterized by an OD_600_ nm of approximately 0.6–0.8. RNA extraction was carried out using the TRIzol method, and sample quality was inspected using a Thermo NanoDrop One and Agilent 4200 Tape Station (36). Approximately, RNA samples were treated with the Epicentre Ribo-Zero rRNA Removal Kit (Illumina) to enrich for mRNA. Sequencing libraries were constructed using the NEBNext Ultra II Directional RNA Library Prep Kit. The constructed libraries underwent a quality inspection before being sequenced on Illumina’s high-throughput sequencing platform with PE150 configuration. The resulting reads were trimmed using fastp v0.23.2 (37) and mapped to the Mab reference genome (NCBI accession number: CP034181). Quantitative analysis of gene expression levels was conducted to identify differentially expressed genes (DEGs) between different samples and to reveal their regulatory mechanisms by combining sequence function information. Gene expression was quantified using RNA sequencing by expectation maximization (RSEM), and fragments per kilobase of transcript per million mapped reads (FPKM) were calculated using Htseq-count (v0.11.2) (38, 39). Differential gene expression analyses were performed using DESeq2 and edgeR (40, 41), with the default screening conditions set to FDR ≤ 0.05 and |log_2_FC (FoldChange)| ≥ 1, applying Benjamini/Hochberg correction for multiple testing.

Functional annotation of the differential gene set was conducted using Gene Ontology (GO) to understand the roles of these genes, metabolic pathways, and other biological processes (42). Additionally, the Kyoto Encyclopedia of Genes and Genomes (KEGG) was used to determine the molecular functional pathways associated with DEGs (43). Functional enrichment analyses, such as GO and KEGG enrichment, were performed using clusterProfiler (44). Furthermore, Rockhopper software was employed for small RNA and transcript structure analysis (45).

### Verification of DEGs by quantitative real-time PCR

RNA extraction and quantitative real-time PCR (qRT-PCR) were performed following the previously described protocol (46). RNA was extracted using the commercial HiPure Bacterial RNA Kit (Magen, Guangzhou, China). The 2^(−ΔΔCT)^ method was used to calculate relative gene expression levels, with *sigA* as a reference gene. GraphPad Prism version 10.3.1 (GraphPad, San Diego, USA) was used to analyze and interpret the results. The RT-PCR experiment was performed with three biological replicates.

### Green fluorescent protein reporter assay

Reporter strains were generated by ectopic expression of green fluorescent protein (GFP) in WT Mab and Δ*sigH,* using the *Np* of *sigH* or the control *hsp60* promoter. This was achieved by cloning *Np* into pMV261-GFP (47)*,* replacing *hsp60*, to construct pMV261-*Np*GFP*,* and then transforming it into WT Mab and Δ*sigH*. The transformants were grown on Middlebrook 7H10 agar plates supplemented with KAN at 100 µg/mL for 3–5 days at 37°C and verified by PCR and sequencing (Fig. S5). Thus, the following GFP reporter strains were constructed in this study: WT: *Np-*GFP (the WT strain ectopically harboring *Np*-GFP), WT: *hsp60-*GFP (the WT strain ectopically harboring *hsp60*-GFP), Δ*sigH: hsp60-*GFP (the Δ*sigH* strain ectopically harboring *hsp60*-GFP), and Δ*sigH: Np-*GFP (the Δ*sigH* strain ectopically harboring *Np*-GFP).

Live fluorescence signal was quantified for all strains in 96-well plates at different optical densities. Bacterial strains were grown in Middlebrook 7H9 broth until the log phase and diluted to six different OD_600 nm_: 0.6, 0.3, 0.15, 0.075, 0.0375, and 0.01,857. Live fluorescence was quantified using a FlexStation 3 Multi-Mode Microplate Reader (Molecular Devices, CA, USA), with excitation and emission wavelengths set at 488 nm and 520 nm, respectively. Statistical parameters were normalized with a negative control (7H9 broth without bacteria), analyzed at different OD_600 nm_. For confocal laser scanning microscopy visualization, bacterial cells were grown until the exponential phase and then centrifuged at 12,000 × *g* for 3 minutes to collect the bacterial cells. The collected bacterial cells were washed with PBS-Tween 80 three times and fixed in 4% glutaraldehyde at room temperature for 30 minutes. The fixed cells were recovered by centrifugation and resuspended in PBS-Tween 80. Finally, fixed bacterial cells were mounted on microscope slides, covered with coverslips, and visualized using a Zeiss LSM 800 laser confocal microscope.

### *MAB_4143c, MAB_3388c,* and *MAB_1011c* overexpress in Δ*sigH*

Overexpression strains were constructed by ectopic expression of genes of interest in Δ*sigH* under the influence of the *hsp60* promoter. *MAB_4143c, MAB_3388c,* and *MAB_1011c* were amplified and cloned into pMV261 to construct pMV261-*MAB_4143c,* pMV261-*MAB_3388c,* and pMV261-*MAB_1011c* plasmids. Generated plasmids were transformed into Δ*sigH*. The transformants were grown on Middlebrook 7H10 agar plates supplemented with KAN at 100 µg/mL for 3–5 days at 37°C and verified by PCR and sequencing (Fig. S6). Thus, the overexpression strains OEΔ*sigHMAB_4143c,* OEΔ*sigHMAB_3388c,* and OEΔ*sigHMAB_1011c* were constructed. Finally, all overexpression strains, WT, Δ*sigH,* and CPMab*sigH,* were subjected to drug susceptibility testing (DST) as we previously described.

### Ethidium bromide accumulation assay

The ethidium bromide (EtBr) accumulation assay was conducted as previously described (48) to evaluate the cell envelope permeability of mycobacterial strains. Mycobacterial cultures were grown in Middlebrook 7H9 medium at 37°C until reaching the mid-log phase. Bacterial suspensions were then normalized to an OD_600_ nm of 0.8 in phosphate-buffered saline (PBS) supplemented with 0.8% glucose. EtBr was added to the wells at a final concentration of 2 μg/mL along with 0.4% glucose. Fluorescence measurements were taken using a FlexStation 3 Multi-Mode Microplate Reader (Molecular Devices, CA, USA), with excitation and emission wavelengths set at 530 nm and 590 nm, respectively. The fluorescence data from EtBr accumulation were recorded at 60-second intervals over a period of 60 minutes at 37°C. Data analysis and plotting were performed using GraphPad Prism version 10.3.1 (GraphPad, San Diego, USA).

### *In vitro* growth rate detection of Mab strains

Bacterial growth rate and viability were observed by measuring changes in absorbance at 600 nm and by assessing CFU/mL. Mab strains were cultured in 7H9 broth until reaching the logarithmic phase. Subsequently, bacterial cultures were diluted to an OD_600_ of approximately 0.015 in individual 100 mL flask bottles. These bottles were then incubated in a shaking incubator set at 200 rpm for 72 hours to assess bacterial growth via OD_600_ measurements and to enumerate CFU/mL. Bacterial proliferation and viability were monitored by periodically measuring OD_600_ using a spectrophotometer every 6 hours. The serially diluted bacteria (100 µL) were plated onto 7H10 agar plates to facilitate the observation of morphological differences among the various strains. Following incubation at 37°C for a period ranging from 3 to 5 days, the plates were examined, and individual colonies were counted. The CFU/mL was calculated employing the formula: CFU/mL = (number of colonies/volume of bacteria plated [in mL]) × dilution factor.

### Scanning electron microscopy

Samples were prepared by growing the bacteria in Middlebrook 7H9 broth until OD_600_ of 0.6–0.8. The bacterial suspension was centrifuged and washed three times with PBS buffer. After washing, bacterial cells were fixed with 4% glutaraldehyde at room temperature for 1 hour. The fixed bacterial cell suspension was centrifuged to eliminate glutaraldehyde and rinsed three times with PBS buffer. Following that, the cells were sequentially dehydrated in a graded series of ethanol solutions 70%, 80%, 90%, and 100%. All the sample specimens were dried using the critical point drying method. The dried specimens were then sputter-coated and visualized under a Gemini SEM 360 scanning electron microscope (ZEISS, Germany) to observe cell length and morphology. Cell length was measured using Nano Measurer (version 1.2), and GraphPad Prism version 10.3.1 (GraphPad, San Diego, USA) was used for data presentation and analysis.

### Molecular docking

Molecular docking was conducted using the AutoDock 4.2.6 (49). The crystal structure of Mtb σ^H^-RNAP (σ^H^-RPo) in complex with promoter DNA (PDB ID: 5zx3, resolution=2.751 Å) was retrieved from the RCSB Protein Data Bank (50). Protein preparation was performed using the Protein Preparation Wizard module in Discovery Studio (BIOVIA), including removal of solvent molecules, addition of hydrogen atoms, correction of missing loops, and retention of the zinc ion cofactors. The optimization was performed with ionization states at pH 7.4 to be consistent with experimental conditions and to mimic physiological conditions. Then, the protein structure was minimized using the OPLS_2005 force field to relieve structural strain. The protein structure grid box was generated around the co-crystallized promoter DNA central coordinates with dimensions of 15 × 15 × 15 Å. The prepared receptor grid of σ^H^-RPo was used in the molecular docking study.

## RESULTS

### Identification of the stress response regulator SigH in Mab

Mab is an opportunistic pathogen that causes significant morbidity in patients with underlying lung disease, including cystic fibrosis (CF). Condition-specific transcriptomic profiling of Mab exposed to synthetic CF sputum medium revealed strong induction of multiple sigma factors and their cognate anti-sigma factors, implicating these regulators in host-associated stress adaptation (51). Sigma/anti-sigma systems are central controllers of bacterial stress responses (26), and their CF-specific upregulation (51) suggested a possible role in pathogenesis and antimicrobial tolerance. These findings inspired us to investigate whether sigma and anti-sigma factors play a role in resistance to multiple antibiotics in Mab. Thus, we identified two adjacent sigma and anti-sigma factors, *sigH* (*MAB_3543c*) and *rshA* (*MAB_3542c*), respectively, for this investigation. We subsequently generated a *rshA* knockout strain (Δ*rhsA*) to study the role of the *rshA* gene in drug resistance. The difference in drug susceptibility between WT and Δ*rhsA* was not significant (data not shown). Notably, the *rshA* gene and *sigH* lie in the same cluster. Mab SigH shares 84% amino acid identity with Mtb SigH (Fig. S7). The stress response factor SigH plays a crucial role in regulating responses to heat and oxidative stress and has been extensively studied in both Mtb and Msm (26). Overexpression of *sigH* in WT (OEMab*sigH*) resulted in a significant increase in resistance to TIG and FQs (Table 1).

**TABLE 1:** MICs of the different Mab strains^*a*^

Antibiotics^*b*^	MIC^*c*^ (µg/mL)/Mab strains^*d*^						
	WT	Δ*sigH*	CPMab*sigH*	CPMtb*sigH*	OEMab*sigH*	CP*hsp60*Mab*sigH*	CP*Np*Mab*sigH*
TIG	4	0.5	4	2	16	4	8
TET	128	32–64	128	64	˃128	128	˃128
CLA	4–64	4–64	4–64	4–64	4–64	4–64	4–64
CLF	4	4	4	4	4	4	4
VAN	128	64	128	128	>128	>128	128
AMK	16	2	16	16	32	16	16
MFX	8	2	8	8	32	32	32
LFX	16	2	16	16	32–64	32	32
IMP	32	32	32	32	32	32	32
CFX	32	32	32	32	32	32	32
RIB	8	2	8	8	16	8	8
LZD	64	64	64	64	64	64	64

^*a*^ The plates were incubated at 37°C for 3 days, except for CLA, which was incubated for 14 days before final reading.

^*b*^ Antimicrobial agents: TIG, tigecycline; TET, tetracycline; CLA, clarithromycin; CLF, clofazimine; VAN, vancomycin; AMK, amikacin; LFX, levofloxacin; MFX, moxifloxacin; IMP, imipenem; CFX, cefoxitin; RIB, rifabutin; and LZD, linezolid.

^*c*^ MIC is defined as the lowest concentration of a drug that inhibits visible bacterial growth.

^*d*^ WT, wild-type Mab; Δ*sigH*, *sigH* deletion strain; CPMab*sigH*, complemented strain expressing Mab*sigH*; CPMtb*sigH*, complemented strain expressing Mtb*sigH*; OEMab*sigH*, wild-type Mab overexpressing Mab*sigH*; **CP*hsp60*Mab*sigH*, complemented strain expressing Mab*sigH* under *hsp60* promoter; **CP*Np*-Mab*sigH*, complemented strain expressing Mab*sigH* under its *Np* promoter.

### Deletion of *sigH* increases Mab hypersensitivity to multiple antibiotics

**Fig 1:**
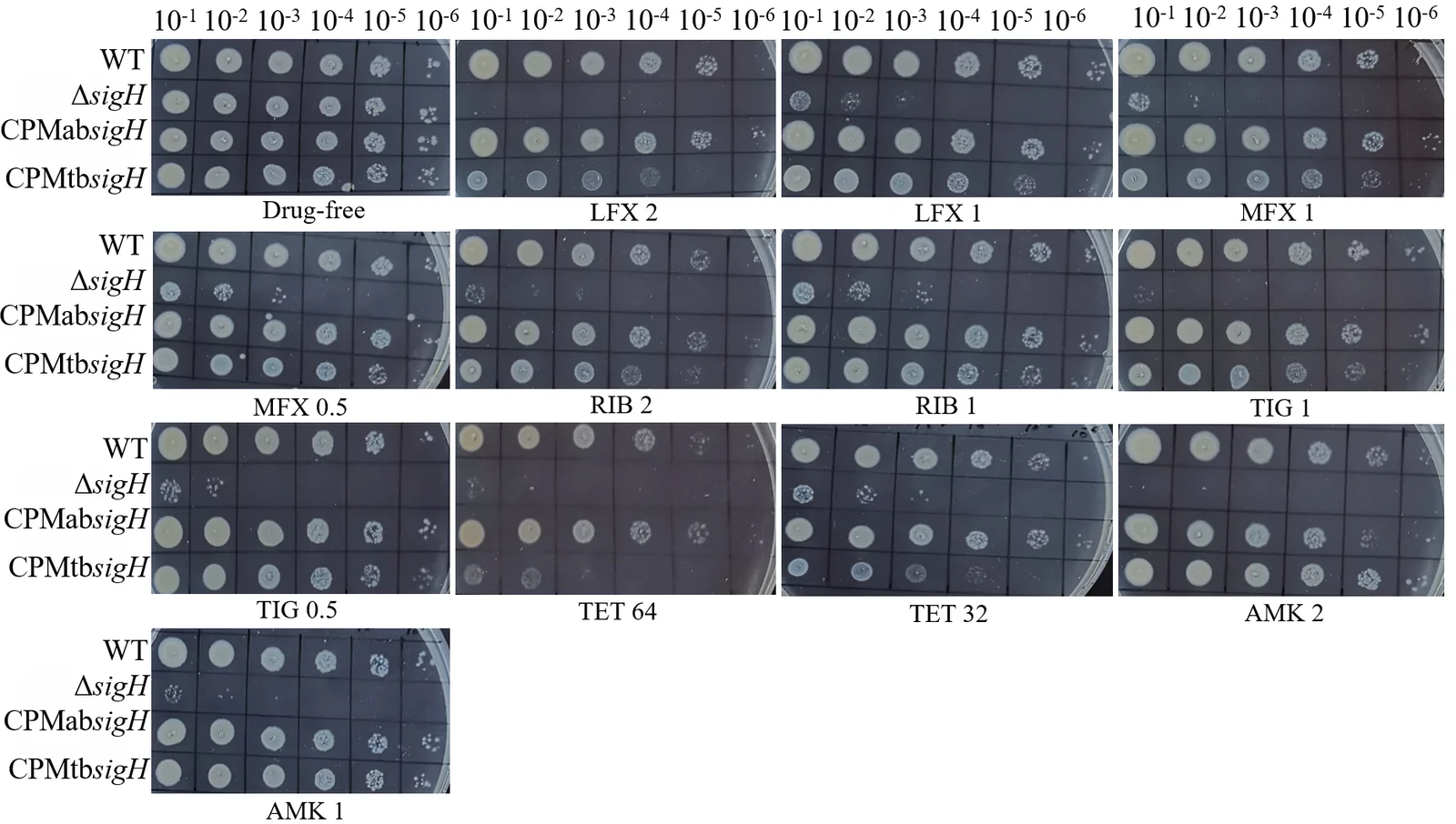
Susceptibilities of different Mab strains to different antibiotics on 7H10 agar plates. Strains were propagated in 7H9 medium at 37°C until an OD_600_ nm of 0.6 was reached. Subsequently, 10-fold serial dilutions were applied and aliquoted onto plain Middlebrook 7H10 agar (drug-free serving as a control) alongside plates supplemented with varying drug concentrations (µg/mL). Following a 3-day incubation at 37°C, the plates were examined. TIG, tigecycline; TET, tetracycline; AMK**,** amikacin; LFX**,** levofloxacin; MFX**,** moxifloxacin; and RIB**,** rifabutin.

To investigate the role of *sigH* in multiple drug resistance, we constructed an in-frame deletion strain of Mab for *sigH* (Δ*sigH*) using CRISPR/Cpf1-assisted recombineering. The sensitivity of WT, Δ*sigH*, and its complemented strains was assessed via broth microdilution and spot growth inhibition on agar plates. Drug susceptibility testing revealed that Δ*sigH* exhibited increased sensitivity to multiple antibiotics, including ribosome-targeting agents such as (TIG, TET, AMK), cell wall synthesis inhibitors (VAN), FQs (LFX, MFX), and RIB. To further verify the role of *sigH* in drug resistance in Mab, complemented strains were constructed by reintroducing Mab *sigH* or its homolog from Mtb (Mtb *sigH*). The complemented strains restored resistance to the tested drugs (Fig. 1; Table 1). Additionally, to confirm the functional role of *sigH* expression level, complemented strains were generated using two constructs: one by expressing Mab*sigH* under its native promoter (*Np*) (CP*Np*Mab*sigH*) and another under the strong mycobacterial *hsp60* promoter (CP*hsp60*Mab*sigH*). Complementation under either promoter restored the drug-resistance phenotype (Table 1). These results collectively demonstrate that SigH contributes significantly to the intrinsic multidrug resistance of Mab, likely through regulation of stress-responsive pathways that enhance bacterial tolerance to diverse antibiotics.

### Deletion of *sigH* influences stress responses in Mab

**Fig 2:**
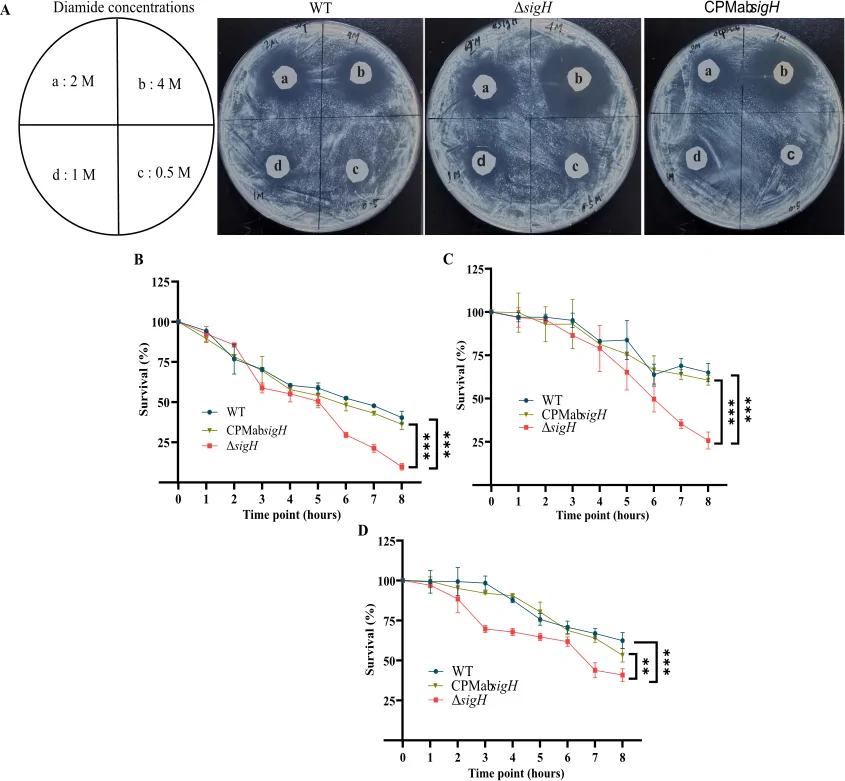
Sensitivity and survival rates of WT, Δ*sigH*, and CPMab*sigH* under stress conditions. Sensitivity of WT, Δ*sigH*, and CPMab*sigH* to different concentrations (M) of diamide (**A**) as well as their survival in the presence of diamide (50 mM) (**B**), H_2_O_2_ (50 mM) (**C**), and heat (45°C) (**D**).

SigH is a crucial regulator of a large transcriptional network that responds to heat and oxidative stress in Mtb (52). It plays an important role in virulence in animal infection models and in responding to extracellular stresses and intracellular survival (53). To investigate the role of *sigH* in Mab stress responses, we conducted heat stress assays and various oxidative stress assays. The diamide induction assay revealed that the Δ*sigH* strain is highly sensitive to thiol-specific oxidation by diamide. This sensitivity was evident from a larger inhibition zone in the Δ*sigH* strain (35 mm) compared to WT (20 mm) and CPMab*sigH* (26 mm) (Fig. 2A; Table S2). After 8 hours, the percentages of survivors for WT, Δ*sigH*, and CPMab*sigH*, respectively, under the different conditions, were as follows: Diamide (40.38%, 9.71%, and 36.25%), H₂O₂ (64.99%, 25.74%, and 60.55%), and heat (62.40%, 40.90%, and 53.26%) (Fig. 2B through D).

### Transcriptomic profiling reveals differential gene expression and correlative patterns among WT, Δ*sigH*, and CPMab*sigH* strains

**Fig 3:**
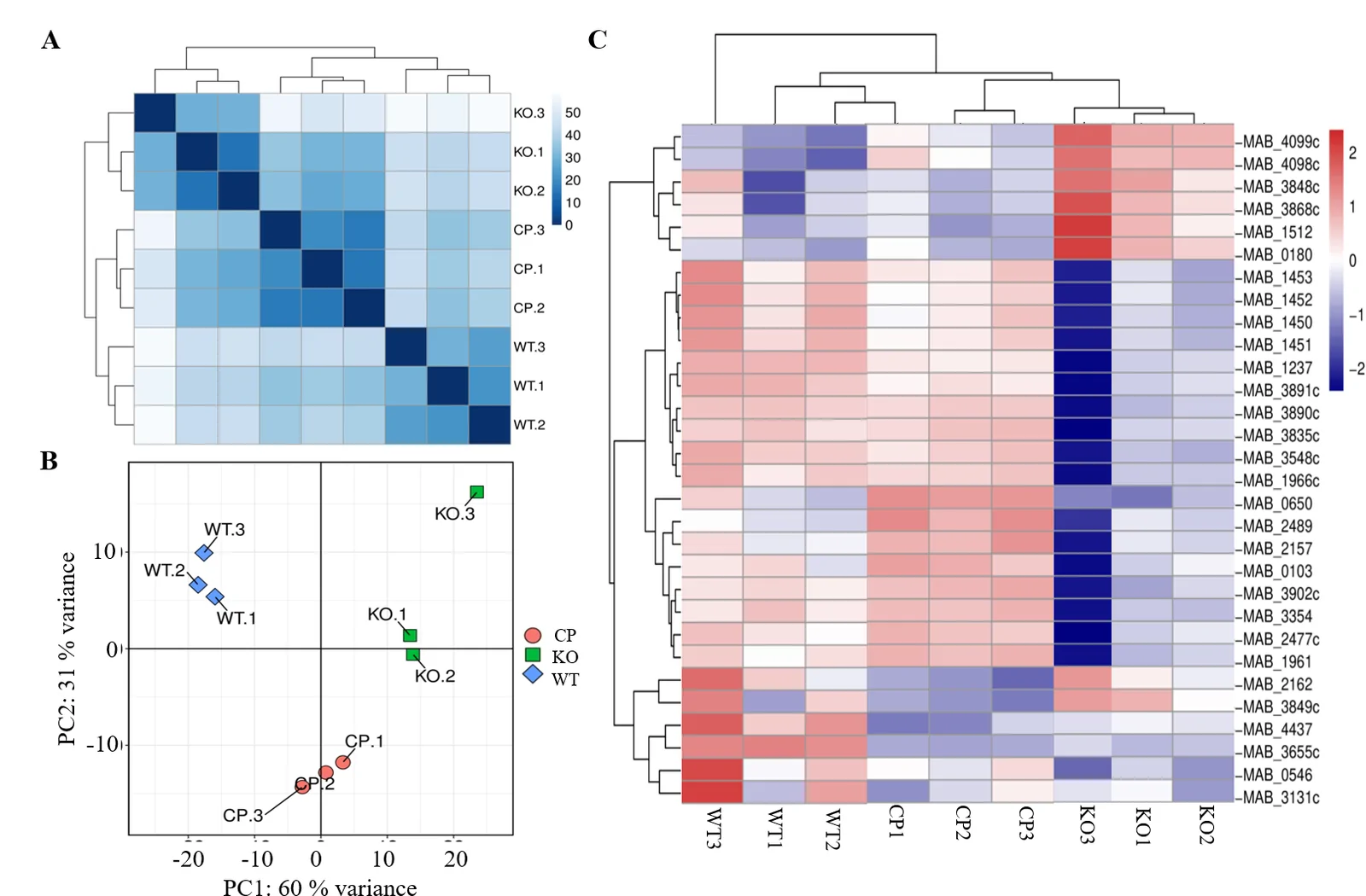
Identification of differentially expressed genes and analysis of their correlations among WT, Δ*sigH*, and CPMab*sigH*. Hierarchical clustering based on the expression of all genes can accurately reflect the relationships among samples (**A**). The first principal component (PC1) and the second principal component (PC2) are plotted in a two-dimensional coordinate graph, with the values on the axis labels representing the percentage of the total variance explained by each principal component (**B**). Based on gene expression data, we performed hierarchical clustering analysis to explore relationships between samples and genes. In the figure, each column represents a sample, and each row represents a gene. Different colors indicate the gene expression levels across various samples. Red indicates higher expression, while blue indicates lower expression. The figure shows the top 30 gene expressions resulting from the clustering analysis (**C**). Note: WT: WT Mab; KO, Δ*sigH*; CP, CPMab*sigH*.

The raw reads of transcriptome data were examined to gain insights into gene expression changes in Δ*sigH*. Several DEGs were found in both Δ*sigH* and CPMab*sigH* when compared to WT. Distance heat maps depicting the expression of all genes were utilized to hierarchically cluster the relationships among samples, thereby accurately reflecting inter-sample relationships (Fig. 3A). Principal component analysis (PCA) was employed to represent the total variance and correlations among the samples (Fig. 3B). This analysis revealed significant differences between Δ*sigH* and both WT and CPMab*sigH*. A correlation heat map was plotted to better understand the DEGs and their relative expression patterns of shared genes among WT, CPMab*sigH*, and Δ*sigH*. The top 30 DEGs are presented in Fig. 3C and Data S1 and S2.

### Deletion of *sigH* affects global gene expression in Mab

**Fig 4:**
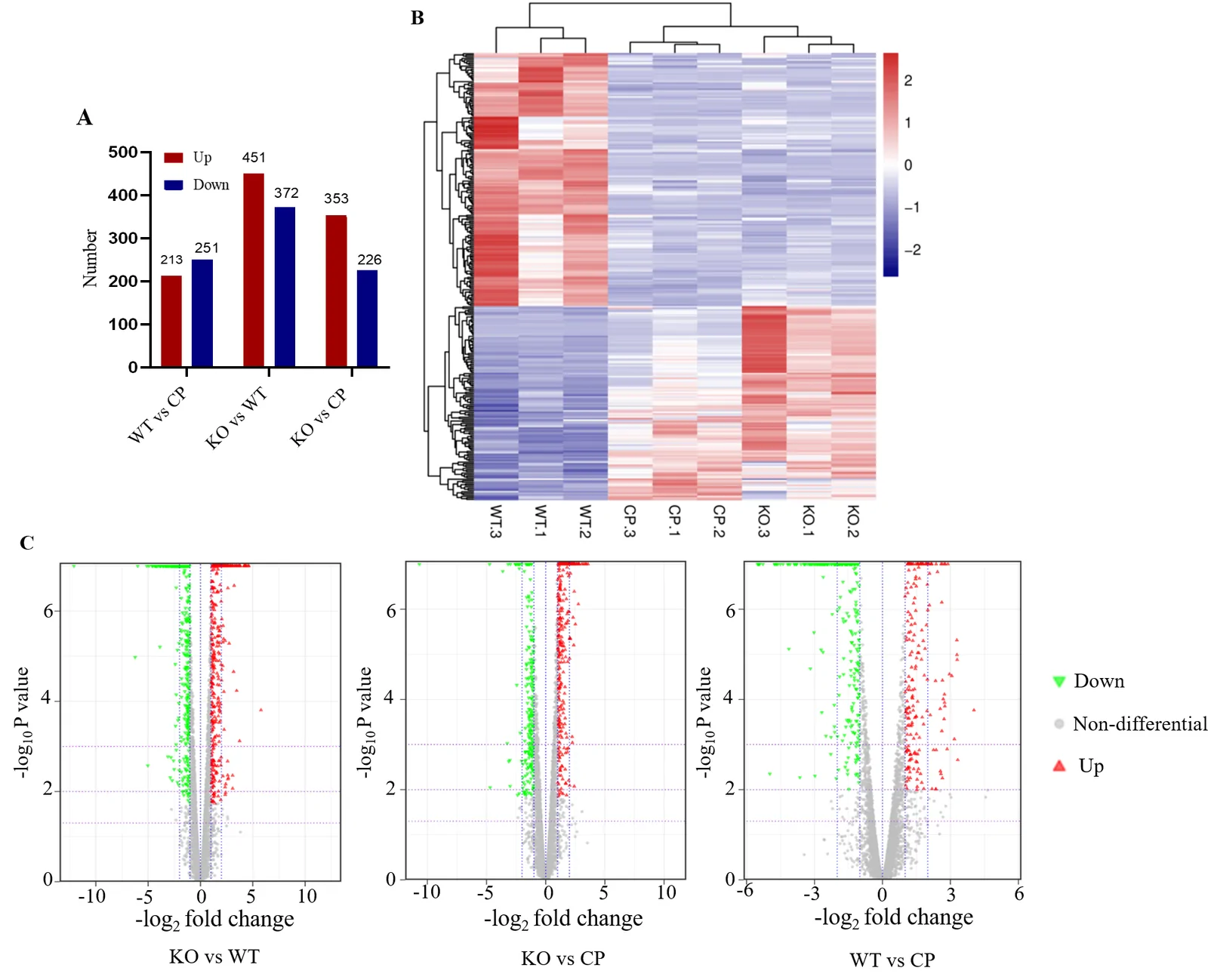
DEGs analysis of different comparison groups. The bar graph illustrates the number of upregulated and downregulated DEGs (**A**). The heat map displays DEGs across different comparison groups; the color intensity indicates the relative expression level of the genes (**B**). The volcano plot presents differentially expressed genes in the comparison groups, with each point representing a gene. The horizontal axis denotes the log_2_ fold change, while the vertical axis represents the negative log_10_ of the *P*-value. Red dots indicate upregulated DEGs, green dots signify downregulated DEGs (downregulated on the left and upregulated on the right), and gray dots represent non-differentially expressed genes (**C**). Only genes with |log_2_FoldChange| > 0 and *P <* 0.05 are included in the volcano plot. Note: WT: WT Mab; KO, Δ*sigH*; CP, CPMab*sigH*.

**Fig 5:**
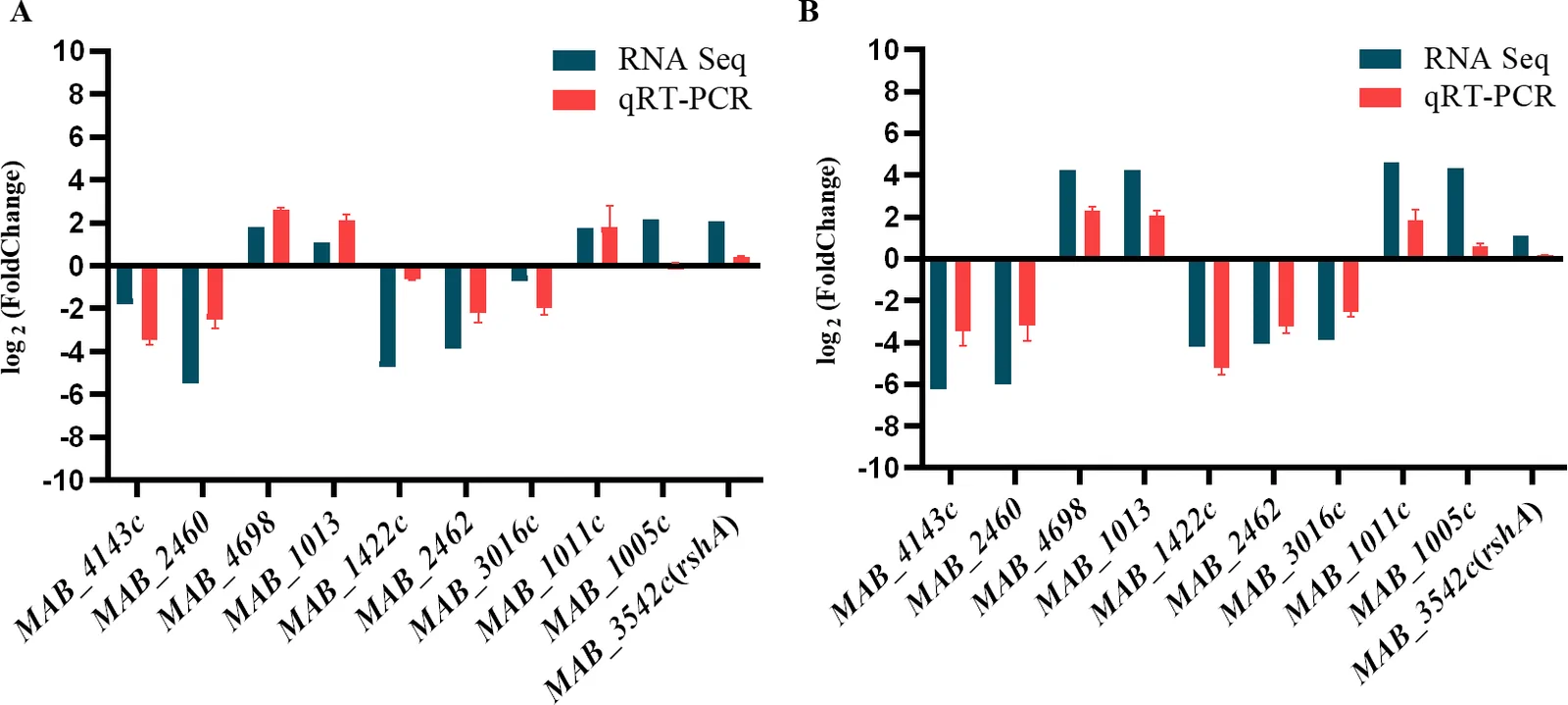
Validation of differential gene expression by qRT-PCR. Relative levels of expression of genes comparison groups are WT vs CPMab*sigH* (**A**) and Δ*sigH* vs WT (**B**). Relative expression of genes was normalized with reference gene *sigA*.

To better understand the phenotype of Δ*sigH*, we identified genes affected by *sigH* deletion. Whole-transcriptome analysis was used to find DEGs in Δ*sigH*. DEGs were analyzed across three comparison groups: Δ*sigH* vs. WT, WT vs. CPMab*sigH*, and Δ*sigH* vs. CPMab*sigH*. We identified 863 DEGs in the Δ*sigH* vs. WT group, with 451 upregulated and 372 downregulated. In the WT vs. CPMab*sigH* group, 464 DEGs were identified (213 upregulated, 251 downregulated). In the Δ*sigH* vs. CPMab*sigH* group, 579 DEGs were identified (353 upregulated, 226 downregulated) (Fig. 4A). The highest number of DEGs was observed in the Δ*sigH* vs. WT group. Notably, 12 genes exhibited the lowest expression levels in the Δ*sigH* vs. WT group compared to the WT vs. CPMab*sigH* group (Table 2; Data S3). These genes include *MAB_4143c*, *MAB_1362*, *MAB_4735*, *MAB_3016c*, *MAB_4694c*, *MAB_2462*, *MAB_4234c*, *MAB_4122*, *MAB_2461*, *MAB_4843*, *MAB_2459*, and *MAB_2460*, encoding putative extracytoplasmic function (ECF) anti-sigma factor, starvation-induced DNA protecting protein/Ferritin and DNA-binding proteins under starvation, probable alternative RNA polymerase sigma factor, glycosyltransferase, sulfonate ABC transporter periplasmic protein, reduced flavin mononucleotide (riboflavin 5′-phosphate) (FMNH_2_) utilizing oxygenase, acyl-CoA dehydrogenase, and many conserved hypothetical proteins. A high increase in the expression of putative YrbE and MCE family proteins was observed in Δ*sigH* compared to WT (Table 2), while several putative sigma and anti-sigma factors (*MAB_3028*, *MAB_3388c*, *MAB_3016c*, *MAB_3549c*, *MAB_3548c*, *MAB_3546c*, *MAB_3542c*, *MAB_3539c*, and *MAB_3538*) were downregulated (Table S3). Other DEGs in the Δ*sigH* vs. WT group are listed in supplementary materials (Data S3). Global differential gene expression across different comparison groups is visualized as a heatmap (Fig. 4B) and a volcano plot (Fig. 4C). We randomly selected 10 genes for validation of the RNA-seq data by qRT-PCR. Though there are differences between RNA-seq and qRT-PCR data in terms of expression levels, there is consistency in whether the genes were upregulated or downregulated (Fig. 5). These results suggest that SigH plays a significant role in shaping global gene expression in Mab.

**TABLE 2:** The most significant alterations in the transcriptome of DEGs across various Mab groups identified using log_2_ fold change values

Gene names	Δ*sigH* vs. WT		WT vs. CPMab*sigH*		Δ*sigH* vs. CPMab*sigH*		Description
	Log_2_F	*P* value	Log_2_F	*P* value	Log_2_F	*P* value	
*MAB_3543c*	−12.100632	1.6E-100	−5.51448	1.06E-39	−10.675	2.476	RNA polymerase sigma-H factor
*MAB_4143c*	−6.243253	0.0,000105	−1.78279	0.013735	−4.7424	5.06E-19	Putative anti-ECF sigma factor
*MAB_2460*	−5.99397	2.80E-53	−5.46303	5.68E-48	−0.53168	0.33503	Conserved hypothetical protein
*MAB_2459*	−5.13153	2.15E-19	−4.76307	1.05E-17	−0.36864	0.68119	Conserved hypothetical protein
*MAB_4843*	−5.0296	0.002647	−4.97085	0.00448	−5.13E-15	1	Hypothetical protein
*MAB_2461*	−4.59441	3.04E-50	−4.62196	9.45E-50	0.028143	1	Putative sulfate ABC transporter, ATP-binding protein
*MAB_4122*	−4.39794	6.07E-14	−3.80961	6.29E-12	−0.58931	0.43792	Putative amino acid permease
*MAB_4735*	−4.3007	2.58E-20	−1.32424	0.000,183	−2.98435	4.84E-10	Putative starvation-induced DNA protecting protein/Ferritin and Dps
*MAB_4234c*	−4.2974	7.30E-19	−3.88915	5.70E-17	−0.40856	0.595278	Putative reduced flavin mononucleotide (riboflavin 5′-phosphate) (FMNH_2_)-utilizing oxygenase
*MAB_4698*	4.268768	6.69E-20	1.814478	0.000355	2.454416	5.48E-11	Conserved hypothetical protein
*MAB_1013*	4.265	4.00E-36	1.105148	0.001335	3.160641	3.36E-25	Hypothetical protein
*MAB_2463*	−4.2640	6.39E-25	−4.50564	1.56E-26	0.24279	0.68209	Putative sulfonate ABC transporter, permease
*MAB_4694c*	−4.2101	1.21E-17	1.418574	5.91E-05	2.806599	6.84E-18	Glycosyltransferase
*MAB_1422c*	−4.2101	1.21E-17	−4.70221	2.94E-20	0.493679	0.372983	Putative acyl-CoA dehydrogenase
*MAB_1060*	−4.20909	6.68E-24	−4.67717	2.85E-26	0.470252	0.378182	Putative alkanesulfonate monooxygenase
*MAB_2218*	−4.19537	1.64E-48	−4.39032	4.67E-51	0.19542	0.561673	Sulfonate ABC transporter periplasmic sulfonate-binding protein SsuA
*MAB_2462*	−4.05529	4.97E-44	−3.8795	6.71E-41	−0.17558	0.653031	Putative sulfonate ABC transporter, periplasmic protein
*MAB_3016c*	−3.89406	2.23E-17	−0.72556	0.033875	−3.17448	2.67E-12	Conserved hypothetical protein
*MAB_3574c*	5.7915	0.000157	4.651039	0.010345	1.086654	0.169225	3-oxoacyl-[acyl-carrier-protein] synthase II
*MAB_4123*	−4.921	1.56E-40	−5.42211	3.24E-43	0.503349	0.430105	Probable monooxygenase
*MAB_1011c*	4.6210	2.29E-49	1.785083	3.79E-09	2.835979	4.02E-25	Putative YrbE family protein
*MAB_1012c*	4.60967	3.76E-30	2.002296	1.05E-06	2.607384	1.11E-14	Putative YrbE family protein
*MAB_4908c*	4.60967	1.93E-16	0.123578	0.66868	−4.74241	5.06E-19	Putative luciferase-like oxidoreductase
*MAB_0219*	2.74E-72	6.31E-69	−4.0746	4.56E-61	−0.49105	0.035696	Conserved hypothetical protein
*MAB_4696c*	4.477659	3.94E-24	2.729194	1.02E-09	1.748204	5.92E-07	Possible methyltransferase
*MAB_1005c*	4.350183	4.31E-26	2.184348	9.10E-09	2.165824	4.61E-09	Putative MCE family protein
*MAB_1009c*	4.326305	7.32E-30	1.559564	9.28E-06	2.766767	1.89E-15	Putative MCE family protein
*MAB_1010c*	4.207413	1.17E-39	0.919939	0.002153	3.287571	4.01E-28	Putative MCE family protein
*MAB_1183*	−4.17225	6.48E-10	−5.47564	1.64E-12	1.338647	0.3295	Conserved hypothetical protein (rhodanese-like)
*MAB_4032*	4.149218	7.37E-18	2.619616	7.04E-07	1.528882	6.61E-06	Putative Mce family protein
*MAB_2217*	−3.79178	1.06E-36	−3.77927	3.53E-38	−0.01219	1	Sulfonate ABC transporter 2c ATP binding subunit SsuB

Following the change in global gene expression after *sigH* deletion, we performed KEGG and GO gene enrichment analyses to determine enriched pathways in Δ*sigH*. The enriched pathways included those involved in metabolism, cellular processes, human diseases, and the processing of genetic and environmental information (Data S4 and S5). The distribution of KEGG DEGs across the different comparison groups is as follows: Δ*sigH* vs. WT (45 upregulated, 76 downregulated); Δ*sigH* vs. CPMab*sigH* (57 upregulated, 10 downregulated); and WT vs. CPMab*sigH* (45 upregulated, 76 downregulated). The enriched pathways are detected in Δ*sigH* vs. WT (85 enriched pathways), WT vs. CPMab*sigH* (64 enriched pathways), and Δ*sigH* vs. CPMab*sigH* (72 enriched pathways) groups (Fig. S8; Data S6 and S7). For the KEGG enrichment analysis of DEGs, the DEGs in the Δ*sigH* vs. the WT group were mainly enriched in ABC-type transporters. Furthermore, GO enrichment analysis identified changes in DEGs associated with sigma factor, transporter, cofactor, and transmembrane transporter activities (Fig. S8; Data S5). These results emphasize that *sigH* inactivation has a profound impact on transcriptional, sigma, anti-sigma, oxidative, and ABC transporter-related gene functions.

### Activity of the *sigH* promoter in WT and Δ*sigH* strains

**Fig 6:**
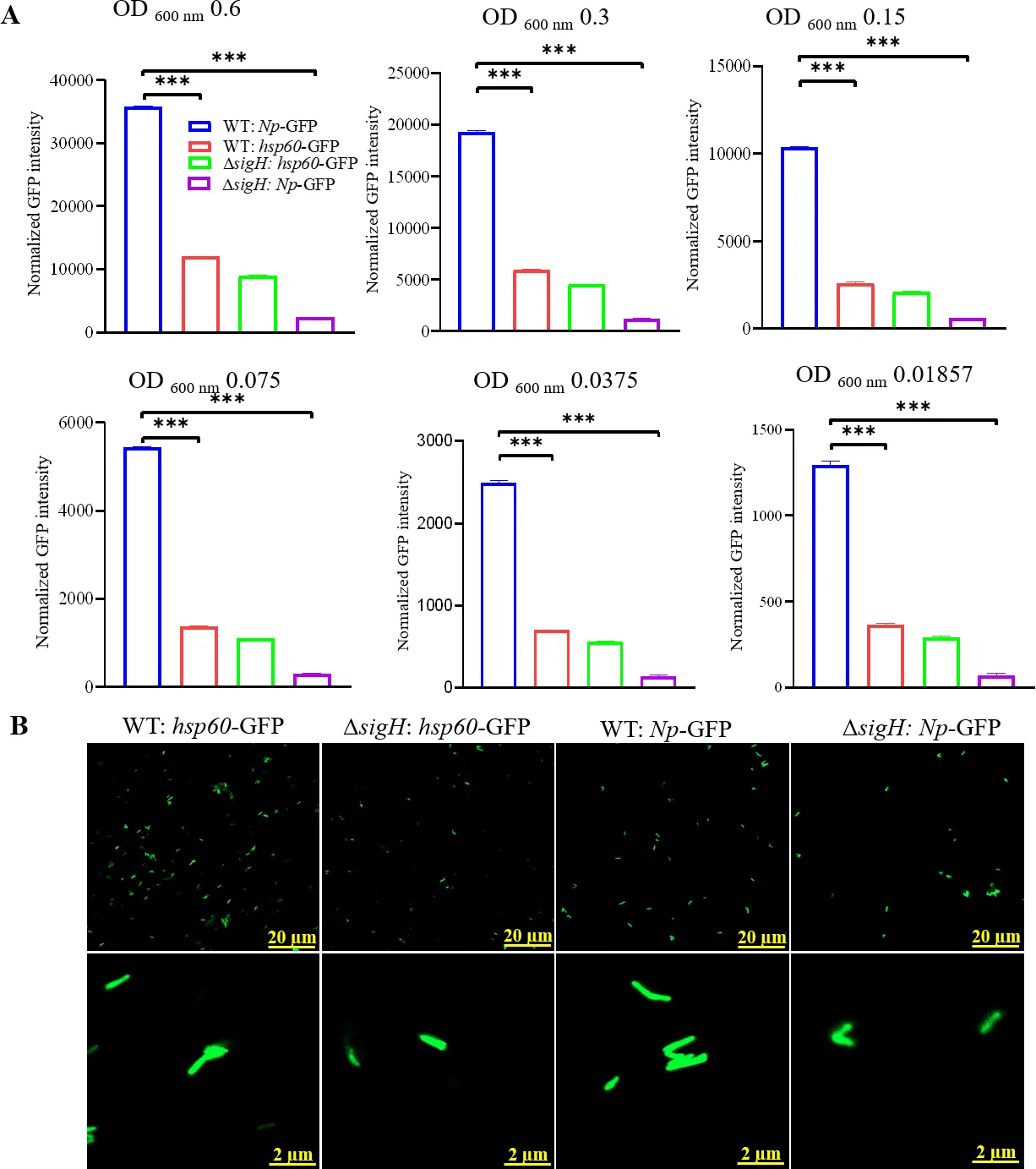
Schematic representation of GFP reporter gene expression under *NP* and *hsp60* promoter and fluorescence intensity detection. GFP with *NP* or *hsp60* expressing WT: *Np-*GFP, WT: *hsp60-*GFP, Δ*sigH: Np-*GFP, and Δ*sigH: hsp60-*GFP strains. GFP live fluorescence signal quantification of different strains. Statistical significance: ns, no significant difference; ***, *P* < 0.001 (**A**). Visualization of GFP-expressing strains by confocal laser scanning microscope (**B**). The bottom part of the picture shows a zoomed-in view of the single cell. WT: *Np-*GFP (the WT strain ectopically harboring *Np*-GFP*)*, WT: *hsp60-*GFP (the WT strain ectopically harboring *hsp60*-GFP), Δ*sigH: hsp60-*GFP (the Δ*sigH* strain ectopically harboring *hsp60*-GFP), and Δ*sigH: Np-*GFP (the Δ*sigH* strain ectopically harboring *Np*-GFP). *Note:* Default laser intensity was used to capture WT: *hsp60-*GFP, Δ*sigH: hsp60-*GFP, WT: *Np-*GFP, and Δ*sigH: Np-*GFP micrographs. However, the Δ*sigH: Np*-GFP signal was very low that the laser intensity had to be increased to get the visible micrograph seen in this figure. Therefore, the live fluorescence data (**A**) were consistent with the GFP signals for all strains under the laser confocal microscope (**B**).

We observed a slight albeit insignificant variation in resistance of complemented strains expressing *sigH* under the influence of either *hsp60* or *sigH Np*, with at least twofold TIG and TET MIC in CP strains expressing *sigH* from *Np* (Table 1). In addition, *Np* may offer a little insight into possible self-regulation by SigH if its activity varies between WT and knockout backgrounds. Therefore, we carried out a GFP reporter assay using either promoter in both WT and Δ*sigH* to assess whether the slight variation in TIG and TET MIC is a random event or likely due to the superior strength of *Np* over *hsp60*, and whether SigH possibly regulates itself. For this, we predicted the promoter upstream of *sigH* using the bacterial promoter prediction web tool BPROM (54). The predicted −10 element (AGGCACAGT) lies within the 5′ UTR of *sigH*, whereas the −35 element (TTGACG) is located within the CDS of the upstream gene (*MAB_3544*), and the two elements have a spacer size of 17 bp. The entire *Np* fragment (5′ UTR + spacer + −35 element) was amplified and cloned directly upstream of the GFP coding sequence in pMVGFP by replacing the *hsp60* promoter. The original pMVGFP construct (with *hsp60* promoter) was used as a control. Interestingly, fluorescence measurements revealed substantially higher quantifiable live GFP expression in WT: *Np*-GFP than in Δ*sigH: Np*-GFP (Fig. 6A), which raises the question of whether SigH self-regulates. In addition, *Np* also appears to show superiority in activity relative to *hsp60* in both WT and knockout backgrounds. All cells produced GFP signals under a laser confocal microscope, though to a lesser level in Δ*sigH: Np*-GFP relative to WT: *Np*-GFP (Fig. 6B). However, the Δ*sigH: Np*-GFP signal was very low, so that the laser intensity had to be increased to get the visible micrograph seen on this figure (Fig. 6B). Therefore, the live fluorescence data were consistent with the GFP signals for all strains under the laser confocal microscope.

**Fig 7:**
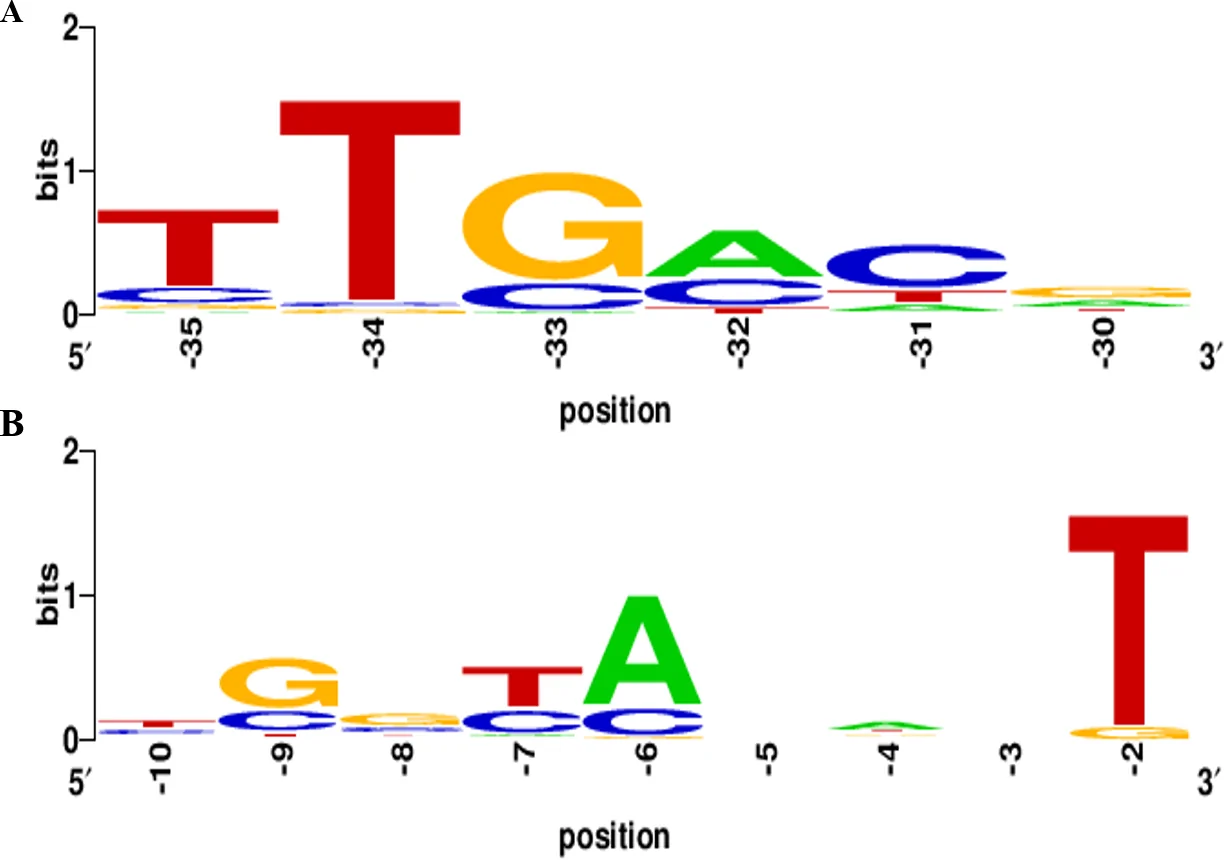
Conserved sequences of predicted promoter elements upstream of *sigH* and selected DEGs. Promoter elements upstream of *sigH* were predicted, then used as reference to align predicted promoter elements of selected DEGs and determine sequence conservation among −35 (**A**) and −10 (**B**) elements of the predicted promoters.

To follow up on the circumstantial evidence of possible self-regulation by *sigH*, we identified all the 862 DEGs in Δ*sigH*, then randomly selected 50 genes (including *sigH*) based on the following criteria: (i) the genes are significantly upregulated or downregulated in Δ*sigH* relative to WT, (ii) presence of a distinct 5′ UTR, and (iii) the presence of both promoter elements, even if one or both of the elements lie(s) within the immediate upstream gene. Twenty-eight of the 50 genes fulfilled the above criteria. As the level of live fluorescence appears to be associated with the presence/absence of SigH, we speculated that SigH likely self-regulates by binding to *Np*. For this reason, we used the *sigH* promoter elements as a reference to carry out sequence alignment and detect consensus or mismatches in promoter elements of those genes that met our set criteria. While there is less consensus among the −10 elements, a high level of consensus is observed in sequences of the −35 elements, with 12 genes having fewer than two mismatches (Fig. 7; Table S4). Ten genes other than the DEGs were randomly selected as a control; their promoters were predicted and aligned to those of *sigH*. All −35 elements in these genes had 2–4 mismatches relative to *sigH* (Table S5). However, lower levels of live fluorescence in Δ*sigH: Np*-GFP (relative to WT: *Np*-GFP) as well as apparent consensus in the sequences of −35 elements of the selected DEGs only constitute circumstantial evidence and would therefore require further experimental validation.

### SigH contributes to drug resistance by influencing other drug resistance determinants in Mab

SigH appears to contribute to drug resistance by influencing other drug resistance determinants in Mab. The multi-drug resistance master regulator WhiB7 has been reported to induce *sigH* transcription, linking SigH to broader stress- and antibiotic-response pathways (21). SigH itself functions as an alternative sigma factor that orchestrates transcriptional programs in response to oxidative, heat, and nitrosative stress and is negatively regulated by its cognate anti-sigma factor, RshA (15). Through these regulatory roles, SigH influences bacterial physiology and gene expression during antibiotic exposure and environmental stress, thereby promoting adaptive tolerance and drug resistance (19, 55, 56).

In this study, transcriptomic analysis revealed that several genes previously implicated in antimicrobial resistance were downregulated in the Δ*sigH* strain compared with WT and CPMab*sigH* strains. These included *MAB_1362*, *MAB_4143c*, *MAB_3028*, *MAB_3388c* (*serB2*), *MAB_3542c* (*rshA*), and *MAB_3016c*. Notably, *MAB_1362* has been associated with intrinsic resistance to AMK, streptomycin (STR), and apramycin (APR) (21). Moreover, the deletion of *MAB_3542c* increases Mab’s sensitivity to TIG (57), while a mutation in *MAB_3388c* (*serB2*) has been linked to cross-tolerance to CFX and MFX (58).

**Fig 8:**
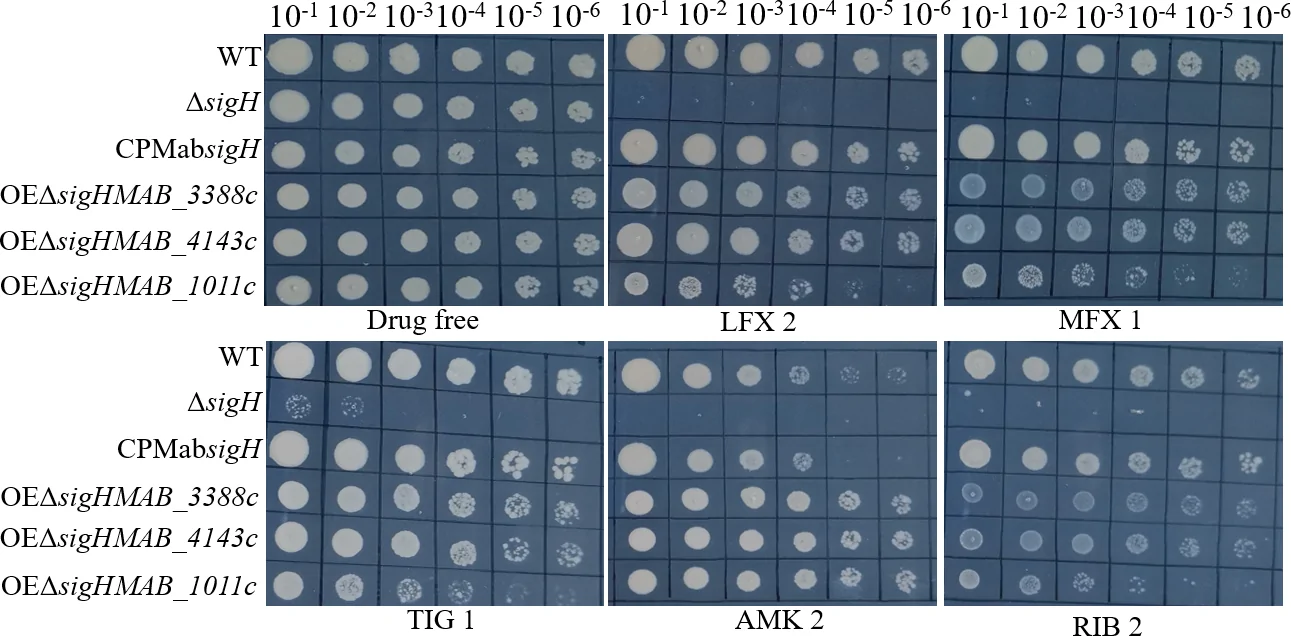
Antibiotic susceptibility profiles of different Mab strains. Strains were propagated in 7H9 medium at 37°C until an OD_600 nm_ of 0.6 was reached. Subsequently, 10-fold serial dilutions were applied and aliquoted onto plain Middlebrook 7H10 agar (drug-free used as a control) alongside plates supplemented with varying drug concentrations (µg/mL). Following a 3-day incubation at 37°C, the plates were examined. TIG, tigecycline, AMK, amikacin; LFX, levofloxacin; MFX, moxifloxacin; and RIB, rifabutin.

To study whether the observed DEGs affect antimicrobial resistance, we selected two downregulated genes (*MAB_3388c* (58), *MAB_4143c*) and one upregulated gene (*MAB_1011c*) and overexpressed them in Δ*sigH*. DST, performed using both broth microdilution and spot growth inhibition assays, demonstrated that overexpression of these genes (OEΔ*sigHMAB_1011c,* OEΔ*sigHMAB_3388c,* and OEΔ*sigHMAB_4143c*) restored the drug resistance phenotype, and in a few cases showed MICs even higher than those of WT and CPMab*sigH* strains (Fig. 8; Table 3). Thus, our results suggest that SigH influences drug resistance in Mab by modulating the expression of other genes directly involved in drug resistance. This regulatory control may enable Mab to adjust stress adaptation and survival under antimicrobial pressure.

**TABLE 3:** MICs of the different Mab strains

Antibiotics^*a*^	MIC^*b*^ (µg/mL)/Mab strains^*c*^					
	WT	Δ*sigH*	CPMab*sigH*	OEΔ*sigHMAB_3388c*	OEΔ*sigHMAB_4143c*	OEΔ*sigHMAB_1011c*
TIG	4	0.5	4	4–2	4–2	2
TET	128	32–64	128	128	>128	˃128
CLF	4	4	4	4	4	4
VAN	128	64	128	128	>128	128
AMK	16	2	16	>128	>128	>128
MFX	8	2	8	8	8	4
LFX	16	2	16	16	32-64	8
CFX	32	32	32	32	32	2
RIB	8	2	8	8	16	4

^*a*^ Antimicrobial agents: TIG, tigecycline; TET, tetracycline; CLF, clofazimine; VAN, vancomycin; AMK, amikacin; LFX, levofloxacin; MFX, moxifloxacin; CFX, cefoxitin; RIB, rifabutin.

^*b*^ MIC is defined as the lowest concentration of a drug that inhibits visible bacterial growth.

^*c*^ WT, wild-type Mab; Δ*sigH*, *sigH* deletion strain; CPMab*sigH*, complemented strain expressing Mab*sigH*; OEΔ*sigHMAB_3388c*, Δ*sigH *overexpressing *MAB_3388c*; OEΔ*sigHMAB_4143c*, Δ*sigH *overexpressing *MAB_4143c*; **OEΔ*sigHMAB_1011c*, Δ*sigH *overexpressing *MAB_1011c*.

### Deletion of *sigH* enhanced Mab cell wall permeability

**Fig 9:**
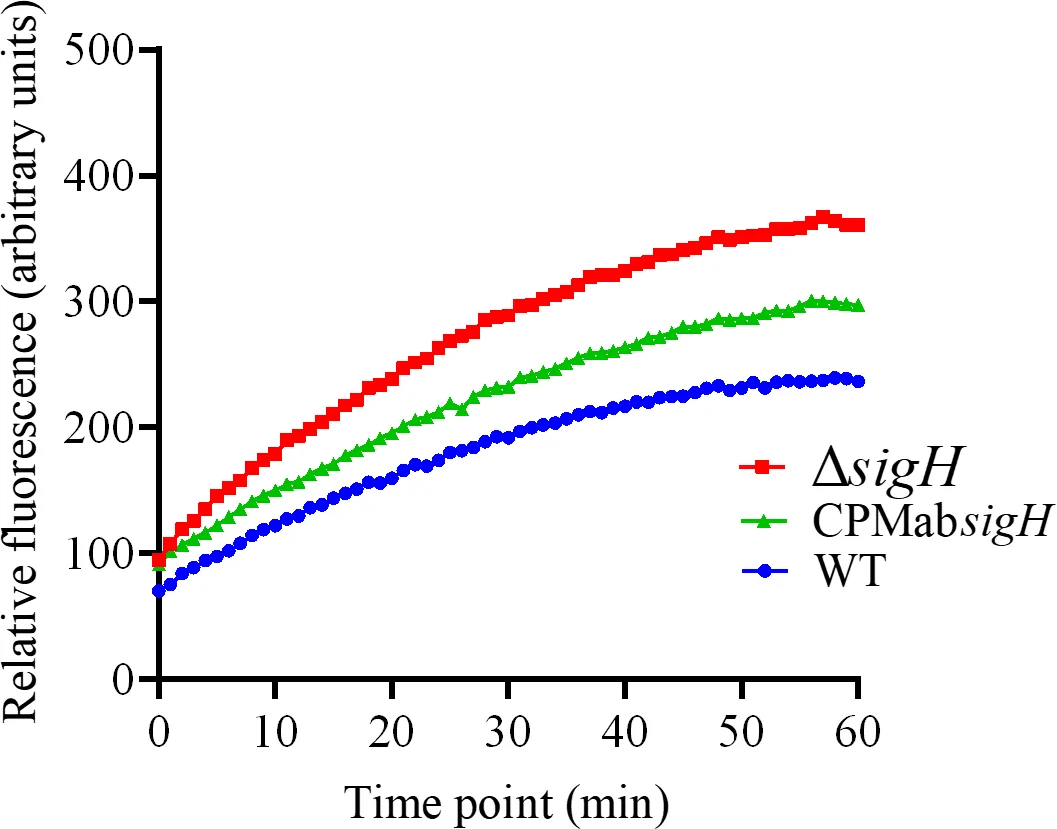
Estimation of cell envelope permeability. Accumulation of EtBr in the cells of WT, Δ*sigH,* and CPMab*sigH* strains.

The deletion of *sigH* affects the cell wall permeability of Mab. To assess this effect, we employed the EtBr accumulation assay, in which the hydrophilic fluorescent dye EtBr intercalates into nucleic acids after traversing the mycobacterial cell envelope (59). Mycobacterial intrinsic resistance to several antimicrobials is thought to be mainly due to decreased cell wall permeability and active efflux mechanisms (59). Therefore, increased intracellular EtBr fluorescence is a sensitive indicator of compromised cell envelope integrity. We observed that compared with the wild-type strain, the Δ*sigH* mutant exhibited significantly higher EtBr accumulation, indicating enhanced permeability of the cell envelope (Fig. 9). Complementation with *sigH* (CPMab*sigH*) partially restored the wild-type phenotype, confirming that the observed increase in permeability was attributable to loss of *sigH* function (Fig. 9). These findings suggest that SigH plays a critical role in maintaining cell wall integrity in Mab, potentially by regulating genes involved in cell envelope biosynthesis, repair, or stress responses.

### Deletion of *sigH* altered bacterial growth and cell length

**Fig 10:**
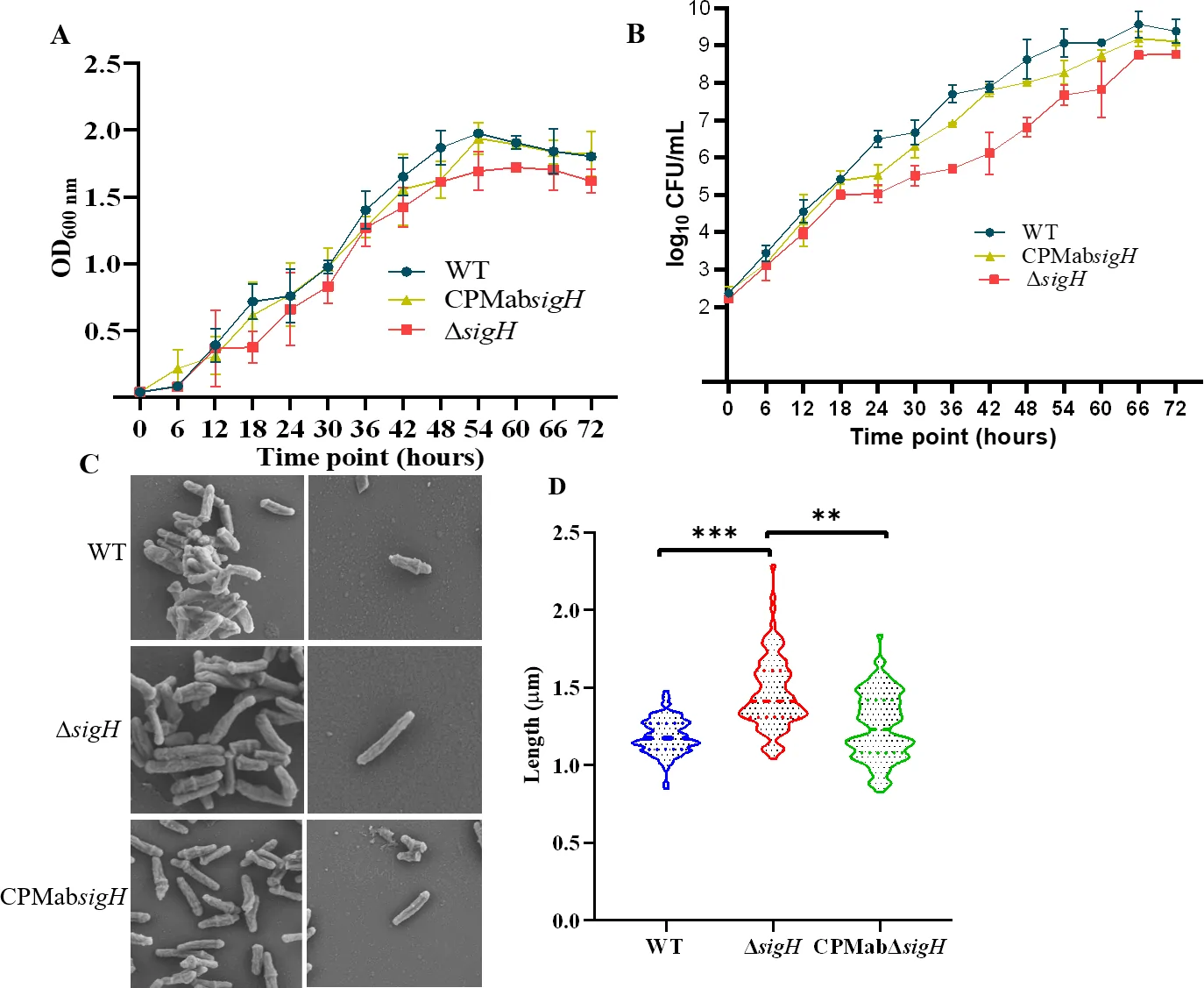
Mab growth rates detection and cell morphology observation. The growth rate of ∆*sigH* compared to WT and CPMab*sigH* is as follows: (**A**) OD_600_ and (**B**) CFU/mL. (**C**) Represents scanning electron microscopy visualization for WT, ∆*sigH,* and CPMab*sigH*. (**D**) Representative graph plot for the measurement of cell lengths and statistical analysis of Mab strains. Statistical significance was determined using an unpaired t-test, with asterisks (** , *P* < 0.01; *** , *P* < 0.0001) indicating significance.

The deletion of *sigH* affects the viability of Mab in different stress conditions. Mab growth rates were checked to determine whether the *sigH* deletion leads to changes under standard *in vitro* conditions. Growth kinetics revealed similar growth patterns for WT, Δ*sigH*, and CPMab*sigH* strains in liquid medium. However, CFU enumeration indicated a modest yet statistically significant reduction in viable counts for the Δ*sigH* mutant compared with WT, whereas the CP strain partially restored the wild-type phenotype (Fig. 10A and B). These outcomes emphasize that sigma factor *sigH* is not critical for maintaining normal growth in Mab, as it has minimal effect on normal bacterial growth.

No obvious differences in colony morphology were observed on solid medium following *sigH* disruption. In contrast, scanning electron microscopy revealed distinct alterations in cell morphology: Δ*sigH* cells were noticeably elongated compared to WT, while complementation with *sigH* (CPMab*sigH*) partially reversed this phenotype (Fig. 10C and D). Quantitative analysis of 100 randomly selected cells from each strain showed average cell lengths of 1.19 µm for WT, 1.46 µm for Δ*sigH*, and 1.25 µm for CPMab*sigH*. The elongation phenotype of the Δ*sigH* mutant implies that SigH influences cell division or envelope homeostasis, consistent with its role as a global regulator of stress-responsive genes (Fig. 4).

### Stable drug–SigH interactions revealed by molecular docking

**Fig 11:**
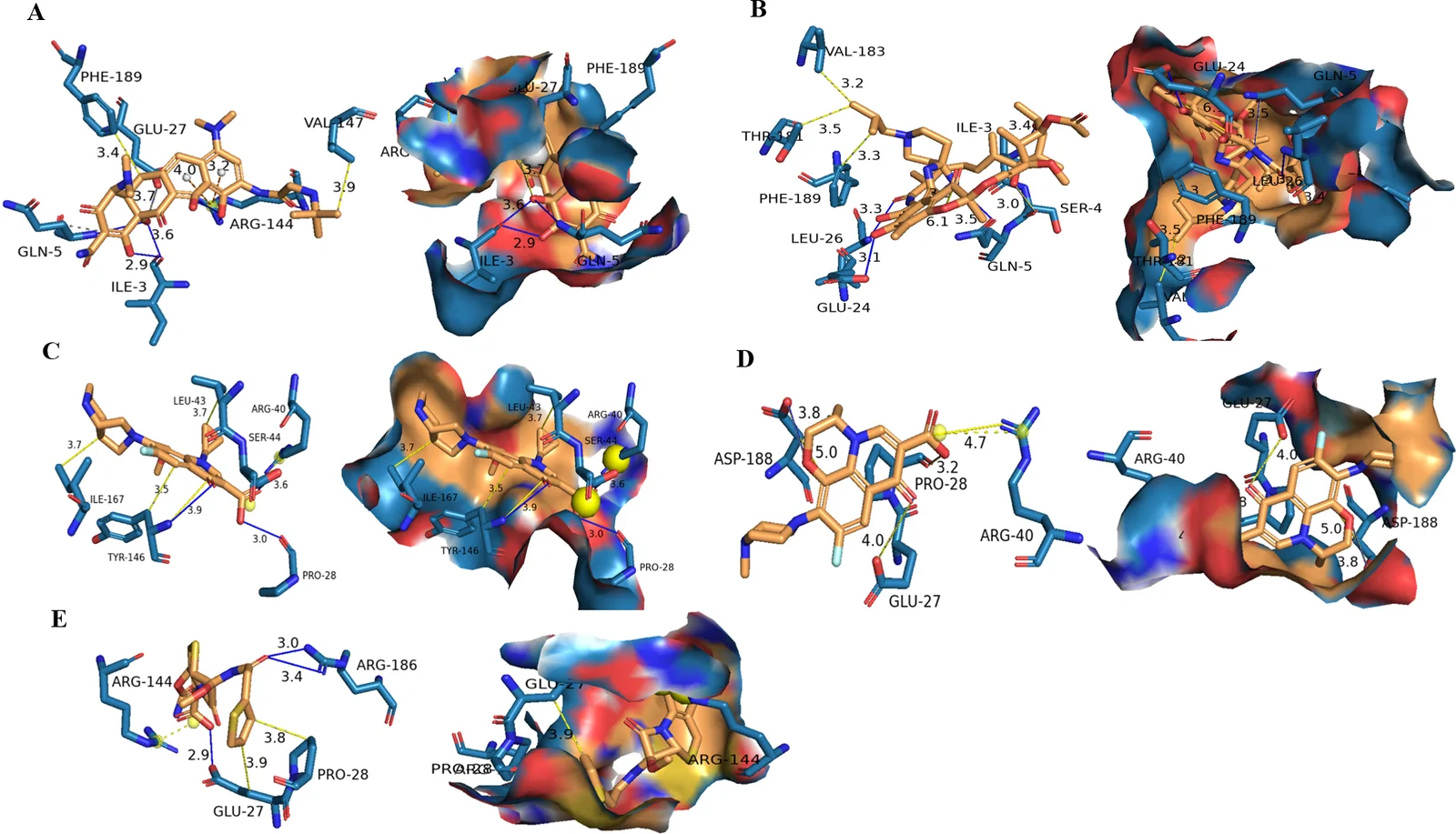
The 2D and 3D structures of the selected five antibiotics (shown as yellow sticks) and SigH protein complex. The binding complex for (**A**) TIG, (**B**) RIB, (**C**) MFX, (**D**) LFX, (**E**) CFX. Strongest binding with multiple hydrophobic bonds and H-bonds. Antimicrobial agents: TIG, LFX, levofloxacin; MFX, moxifloxacin; CFX, cefoxitin; RIB, rifabutin.

Molecular docking studies provide insights into the energetics and structural basis of ligand–protein interactions (60). To explore the potential involvement of SigH in multidrug resistance, we performed molecular docking analyses to evaluate its binding affinity with a panel of clinically relevant antibiotics, including TIG, MFX, LFX, RIB, and CFX. Due to the lack of a crystal structure of SigH from Mab, we used the Mtb SigH homolog as a structural template, which shares 84% amino acid identity with the Mab protein (Fig. S7). Molecular docking outcomes calculated by AutoDock 4.2.6 showed that the lowest value and promising binding affinities were obtained for TIG, MFX, LFX, and RIB to *sigH*, with stable binding energies (kcal/Mol) of –7.72, –7.87, −7.55, and −8.08, respectively (Table 4). The highest interacting residues were obtained for RIB and MFX (Fig. 11). In contrast, CFX displayed weaker and less stable interactions with a binding energy of –3.76 kcal/Mol, consistent with our DST results showing reduced phenotypic resistance to these agents.

**TABLE 4:** Autodock predicted binding energies and RMSD values for antibiotics SigH protein complexes^*a*^

Serial no	Antibiotics	Binding energy (kcal/Mol)	Inhibition constant (Ki)	RMSD	Interacting residues
1	TIG	−7.72	64.15 µM	113.2	ILE-23, GLN-5, GLU-27, ARG-144, VAL-147, PHE-189
2	MFX	−7.87	50.16 µM	88.9	PRO-28, ARG-40, LEU-43, SER-44, TYR-146, ILE-167
3	LFX	−7.55	85.49 µM	91.5	GLU-27, PRO-28, ARG-40, ASP-188
4	RIB	−8.08	35.01 µM	88.7	ILE-3, SER-4, GLN-5, GLU-24, LEU-26, THR-81, VAL-183, PHE-183
5	CFX	−3.76	1.76 mM	116.891	Arg-186, Pro-28, Glu-27, Arg-144

^*a*^ Antimicrobial agents: TIG, tigecycline; LFX, levofloxacin; MFX, moxifloxacin; CFX, cefoxitin; RIB, rifabutin.

## DISCUSSION

The rapidly growing NTM species Mab is an increasingly important opportunistic pathogen whose intrinsic resistance to multiple antibiotics complicates clinical management and contributes to high morbidity and mortality (61, 62). Understanding the molecular basis of its intrinsic resistance is therefore essential for the identification of new therapeutic targets and the development of more effective treatment strategies.

Bacterial pathogens dynamically regulate their gene expression in response to environmental stress during infection, which is crucial for survival and virulence (63). Sigma factors play a key role in this adaptive process by directing RNA polymerase to specific promoter regions (24, 64). Among them, SigH has been established as a major stress-responsive sigma factor in Mtb, Msm, and *Mycobacterium avium ssp. paratuberculosis* (Mav). SigH modulates transcription of genes involved in oxidative and heat stress responses and regulates other extracytoplasmic function sigma factors and redox systems (24, 64). Although the SigH function in Mab is less well characterized, previous studies have linked it to resistance to TIG and AMK (21, 65). Mab SigH shares 84% amino acid identity with Mtb SigH, suggesting functional conservation. In this study, we demonstrated that SigH confers resistance not only to TIG and AMK but also to multiple drugs in Mab (Table 1; Fig. 1). The increased sensitivity of the ∆*sigH* strain to antibiotics such as LFX, MFX, TIG, TET, AMK, VAN, and RIB highlights this role (Table 1; Fig. 1). Additionally, Δ*sigH* showed greater sensitivity to AMK and TIG compared to the previous study (21), and it is not clear whether this stems from strain variation or other factors*.* To ensure the validity of this phenotype, we also performed complementation experiments, which confirmed that the profound sensitivity observed is a genuine and *sigH*-specific phenotype. However, the Δ*sigH* mutant showed significantly increased sensitivity to these antibiotics, while complementation restored resistance, underscoring the conserved role of SigH in mycobacterial drug resistance (16, 17, 66).

In addition, Δ*sigH* exhibited markedly reduced survival under oxidative (H₂O₂), thiol-oxidative (diamide), and heat stress conditions (Fig. 2). These results confirm that SigH is essential for adaptation to multiple stressors, consistent with previous findings in Mtb and Msm. Interestingly, diamide treatment inhibited growth less effectively on agar than in broth, likely reflecting its bacteriostatic rather than bactericidal activity and the influence of exposure duration (67). Bacteria can therefore regain normal growth after diamide exposure. In this study, more growth was regained on agar than in broth, which stems from the fact that the duration of exposure on agar (72 hours) was longer than in broth (8 hours). The ability of Δ*sigH* to resume growth after diamide exposure on agar suggests reversible stress adaptation once thiol homeostasis is restored. Nevertheless, these findings indicate that SigH’s role in stress response is conserved across these mycobacterial species.

Sigma factors are global gene regulators and key transcription activators in mycobacterial pathogenesis. They bind to RNA polymerase, enhancing affinity for specific promoters (68). To investigate *sigH’s* influence on global gene expression in Mab, we performed transcriptomic profiling of WT, ∆*sigH*, and the complemented strain CPMab∆*sigH* (Fig. 3). Significant changes were observed in the gene expression profile of ∆*sigH* compared to WT, while CPMab*sigH* largely restored the WT expression profile (Fig. 3). A large set of genes was differentially expressed: 863 DEGs (451 upregulated, 372 downregulated) in ∆*sigH* vs. WT; 464 (213 upregulated, 251 downregulated) in WT vs. CPMab*sigH*; and 579 (353 upregulated, 226 downregulated) in ∆*sigH* vs. CPMab*sigH* (Fig. 4; Table 2). KEGG enrichment analysis revealed that the most downregulated (log_2_FC ≤ −3) genes in ∆*sigH* were ABC-type transporters (22%), partially explaining the increased antibiotic sensitivity. We validated the differential gene expression data by qRT-PCR of selected DEGs. Though expression levels of the selected DEGs appear to vary between RNA-seq and qRT-PCR, the differential expression pattern (upregulation or downregulation) remained consistent across both methods (Fig. 5).

A promoter ensures that its associated gene is strongly transcribed only when the appropriate sigma factor is present and active (69). Promoter analysis provided further insights into SigH regulatory mechanisms. Here, we predicted the SigH *Np*, amplified it, and used it to express GFP and compared its activity with the strong *hsp60* promoter in both WT and Δ*sigH* backgrounds. GFP fluorescence intensity was approximately 10-fold higher than that in Δ*sigH* (Fig. 6). This clearly indicates that the presence of SigH corresponds to higher live fluorescence, which implies possible binding of SigH to the promoter sequence to produce a higher quantity of live fluorescence in WT relative to Δ*sigH.* In addition, the −35 element of SigH promoter appeared to share a high level of consensus with −35 elements of selected DEGs, with 12 DEGs having a single or even no sequence mismatch (Fig. 7; Tables S4 and S5). The fact that higher sequence consensus was seen among the −35 rather than −10 elements is consistent with the fact that σ^70^-like sigma factors bind to the −35 element to initiate transcription (70). Though this shares some consistency with previous findings (71), further experimental validation is required to show whether SigH binds to the predicted promoter elements.

Downregulation of genes associated with antibiotic resistance was observed, including *MAB_1362* (an alternative RNA polymerase sigma factor), *MAB_3542c* (an anti-sigma factor), and *MAB_3388c* (a phosphoserine phosphatase, *serB2*). Although we did not test sensitivity to STR and APR, these results align with the observed sensitivity profile in previous studies (21, 57, 58). Interestingly, 12 genes were highly upregulated (log_2_FC ≥ 2), including five genes (two *yrbE* and three *mce* genes) within an *mce* operon. MCE proteins are associated with virulence and stress responses in mycobacteria (66). Previous studies suggested that oxidative stress, hypoxia, and nutrient deprivation can modify MCE protein expression, implying a role in stress responses (72–74). Another *mce* gene (*MAB_4032*) was also highly upregulated. To examine whether altered expression of specific DEGs contributes to the drug susceptibility phenotype, we overexpressed two downregulated genes (*MAB_3388c*, *MAB_4143c*) and one upregulated gene (*MAB_1011c*) in Δ*sigH*. Interestingly, all overexpressed strains regained resistance to the drugs, especially showing higher levels of resistance to AMK (Fig. 8). These results suggest that SigH maintains optimal expression levels of several resistance determinants to sustain intrinsic tolerance, while its absence perturbs this regulatory balance. However, gene upregulation, as in the case of *MAB_1011c*, may suggest that in the absence of SigH, the bacteria deploy alternative drug resistance machinery to compensate for lost ability to resist drugs, which, however, appears to be insufficient for Δ*sigH* to maintain wild-type-level resistance. The heightened level of resistance to AMK in the overexpressed strains warrants further investigation into possible compensatory mechanisms.

ABC transporters are crucial mediators of drug efflux in mycobacteria (75–77). The downregulation of ABC-type transporters in Δ*sigH* is not surprising, as it would be reasonable to suspect that disruption of *sigH* likely impairs efflux activity and therefore contributes to reduced drug tolerance in Mab (Table 1; Fig. 1). High accumulation of EtBr in Δ*sigH* relative to WT supports this hypothesis and indicates elevated cell wall permeability (Fig. 9). Complementation partially restored the WT phenotype, confirming SigH’s role in maintaining cell envelope integrity. Although scanning electron microscopy reveals no apparent changes in cell morphology, except that Δ*sigH* cells appear to be longer than WT and CPMab*sigH* (Fig. 10C and D). In addition, we observed no changes in the lipid profiles of all strains (data not shown). Therefore, the increased drug sensitivity of Δ*sigH* likely results from a combination of impaired efflux function, altered cell permeability, and disrupted regulation of drug resistance–associated genes. However, the efflux aspect of this assumption requires further investigation for its validation.

Moreover, our molecular docking analysis suggests that SigH may have the capacity to accommodate interactions with certain antibiotics at the structural level. Though, these computational analysis support our phenotypic drug susceptibility data, however, these predictions require further experimental validation to determine their biological and functional relevance.

A synthetic drug-like molecule, SMARt-420 (Small molecule aborting resistance), has been shown to inhibit the DNA-binding activity of EthR2 (Rv0078), a transcriptional repressor of EthA2 in Mtb. Thereby, SMARt-420 profoundly facilitates the bioactivation and antibacterial effect of ethionamide against Mtb (78). Similarly, inhibition of SigH, a global regulator that extensively controls stress and drug resistance pathways, may offer not only a promising strategy for sensitizing Mab to existing antibiotics but can also be a potential target for new drugs, thus deserving further exploration.

### Summary 

In short, we have identified the role of the mycobacterial sigma factor SigH in stress responses and multidrug resistance in Mab. Deletion of *sigH* resulted in downregulation of multiple resistance determinants, increased cell wall permeability, impaired stress tolerance, and heightened susceptibility to diverse antibiotics. Transcriptomic and functional analyses revealed that SigH orchestrates a broad regulatory network encompassing efflux transporters, stress-response genes, and drug resistance–associated proteins. We propose that inhibition of SigH or disruption of its regulatory interactions could serve as a novel strategy to overcome the intrinsic drug resistance of Mab. Additionally, we suggest that as a compensatory mechanism for this downregulation, the bacteria upregulate the expression of certain proteins, such as YrbE and MCE family proteins. Although these findings require further experimental validation, this study lays the foundation for future mechanistic studies to establish SigH as a potential drug target in Mab.

## SUPPLEMENTAL MATERIAL

Data S1 spectrum.03965-25-s0001.xlsx Hierarchical clustering analysis.

Data S2 spectrum.03965-25-s0002.xlsx FPKM statistical results.

Data S3 spectrum.03965-25-s0003.xlsx DEGs analysis between three different groups.

Data S4 spectrum.03965-25-s0004.xlsx Functional KEGG DEGs analysis.

Data S5 spectrum.03965-25-s0005.xlsx Functional GO DEGs analysis.

Data S6 spectrum.03965-25-s0006.xlsx DEGs KEGG enrichment analysis.

Data S7 spectrum.03965-25-s0007.xlsx Screening of DEGs, KEGG enrichment analysis.

Supplemental material spectrum.03965-25-s0008.docx Fig. S1 to S8; Tables S1 to S5.

ASM does not own the copyrights to Supplemental Material that may be linked to, or accessed through, an article. The authors have granted ASM a non-exclusive, world-wide license to publish the Supplemental Material files. Please contact the corresponding author directly for reuse.
