# Supplementary material for: The stress response factor SigH mediates intrinsic resistance to multiple antibiotics in Mycobacterium abscessus
Source: Microbiol Spectr. 2026 Jun 15;14(7):e03965-25. doi: 10.1128/spectrum.03965-25 (PMC13340314; doi:10.1128/spectrum.03965-25)
Supplement: Supplemental material — Fig. S1 to S8; Tables S1 to S5. [file spectrum.03965-25-s0008.docx]

**The stress response factor SigH mediates intrinsic resistance to multiple antibiotics in *Mycobacterium abscessus***

Md Shah Alam ^a,b,c,d#^, Mst Sumaia Khatun ^a,b,c,d#^, Buhari Yusuf ^a,b,c,d^, Lijie Li ^a,c,d,e^, Aweke Mulu Belachew ^a,b,c,d^, Haftay Abraha Tadesse ^a,b,c,d^, Jingran Zhang ^a,c,d,e^, Xirong Tian ^a,b,c,d,f^, Cuiting Fang ^a,b,c,d,f,g^, Yamin Gao ^a,c,d^, Zhiyong Liu ^f,h^, Jinxing Hu ^g^, Xinwen Chen ^h^, Nanshan Zhong ^c,f,h^, Liqiang Feng^a,b,c,d^, H.M. Adnan Hameed ^a,b,c,d*^, Shuai Wang ^a,c,d,g*^, Tianyu Zhang ^a,b,c,d,f,g,h*^

^a^ State Key Laboratory of Respiratory Disease, Guangzhou Institutes of Biomedicine and Health, Chinese Academy of Sciences, Guangzhou 510530, China

^b^ University of Chinese Academy of Sciences, Beijing 100049, China

^c^ Guangdong-Hong Kong-Macao Joint Laboratory of Respiratory Infectious Diseases, Guangzhou Institutes of Biomedicine and Health, Chinese Academy of Sciences, Guangzhou 510530, China

^d^ China-New Zealand Joint Laboratory on Biomedicine and Health, Guangzhou 510530, China

^e^ School of Life Sciences, University of Science and Technology of China, Hefei 230027, China

^f^ State Key Laboratory of Respiratory Disease, National Clinical Research Center for Respiratory Disease, The National Center for Respiratory Medicine, Guangzhou Medical University, Guangzhou 510230, China

^g^ State Key Laboratory of Respiratory Disease, Guangzhou Chest Hospital, Guangzhou 510095, China

^h^ Guangzhou National Laboratory, Guangzhou 510320, China

**^*^Correspondence:** H.M. Adnan Hameed, [adnan@gibh.ac.cn](mailto:adnan@gibh.ac.cn); Shuai Wang, [wang_shuai@gibh.ac.cn](mailto:wang_shuai@gibh.ac.cn); Tianyu Zhang, [zhang_tianyu@gibh.ac.cn](mailto:zhang_tianyu@gibh.ac.cn).

^＃^These authors contributed equally to this work.

**Supplementary materials**

**
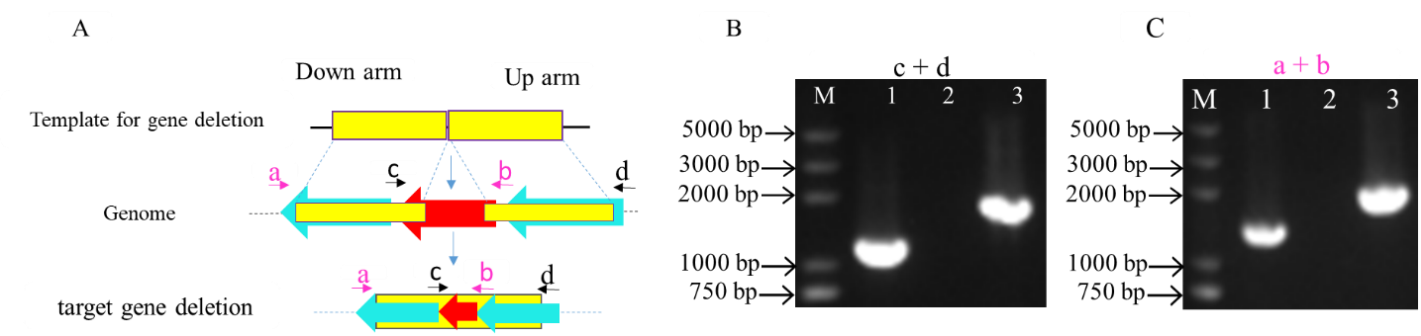
**

**Figure S1:** **Construction of *sigH* deletion strain using CRISPR-Cpf1 assisted recombineering system**.

(A) Schematic diagram of the CRISPR-Cpf1 strategy used to delete the target genes. (B) Δ*sigH* knockout strain confirmation using the primers Id3543c-F/ pMV261- *MAB_3543c* R. M Lane indicated DNA marker, 1 Δ*sigH* knockout strain (1160 bp), 2 negative control (No template), and 3 wild type Mab strain (WT) (1757 bp). (C) Δ*sigH* knockout strain confirmation using the primers Id3543c-R/ pMV261- *MAB_3543c* F. M Lane indicated DNA marker, 1 Δ*sigH* knockout strain (1304 bp), 2 negative control (No template), and 3 wild type Mab strain (WT) (1901 bp). It's confirmed that 597 bp has been deleted.


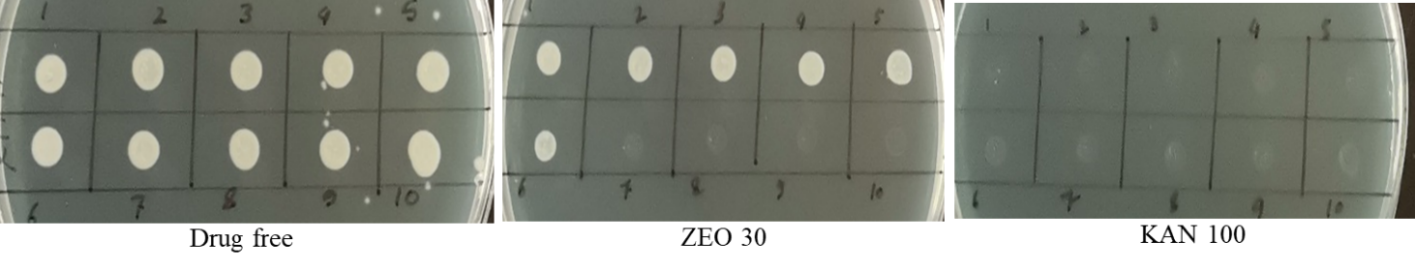


**Figure S2:** **Marker-less Δ*sigH* strain construction confirmation by agar method.** Plates were incubated at 37 ºC and captured images after 3 days. Drug-free plates exhibit colonies. and the Marker-less Δ*sigH* strain lost the marker, which was demonstrated by no observation of bacterial growth in zeocin (ZEO) and kanamycin (KAN)-containing plates, indicating that most colonies lost their marker genes. Drug concentration was used at µg/mL.


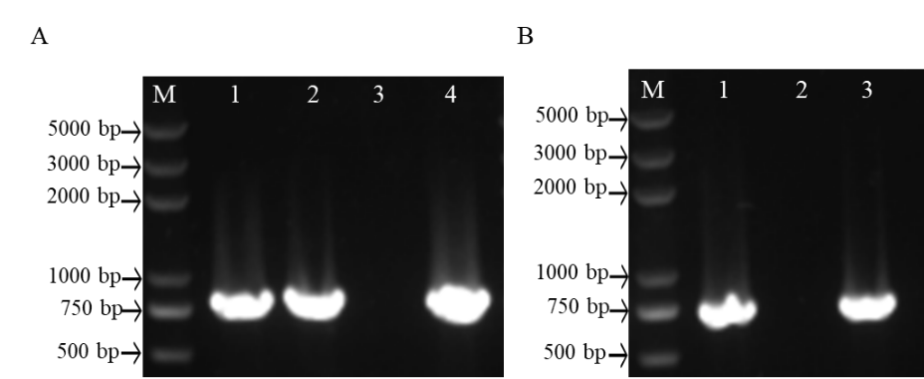


**Figure S3:** **Confirmation of *sigH* Mab overexpressing strains, and complement strains using *sigH* Mab*,* and its homologs, *sigH* Mtb. (**A) Notes: CPMab*sigH* and OEMab*sigH*Mab strains confirmation using the primers JD-pMV261-F/R. Lane M indicates DNA marker, lane 1 CPMab*sigH*, lane 2 OEMab*sigH*Mab strain, lane 3 negative control (Δ*sigH*), and lane 4 positive control (pMV261-*sigH*Mab). The expected length of the fragment is 745 bp. (B) Notes: CPMtb*sigH* strain confirmation using the primers JD-pMV261-F/R. M Lane indicated DNA marker, lane 1 CPMtb*sigH,* lane 2 negative control (Δ*sigH*), lane 3 positive control (pMV261-*sigH*Mtb). The expected length of the fragment is 724 bp.


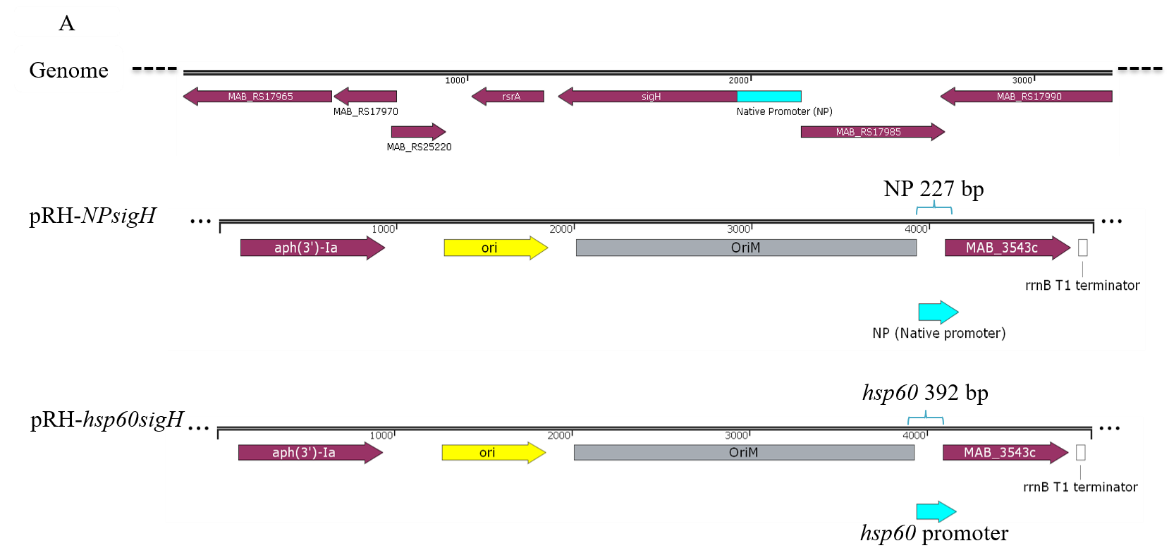


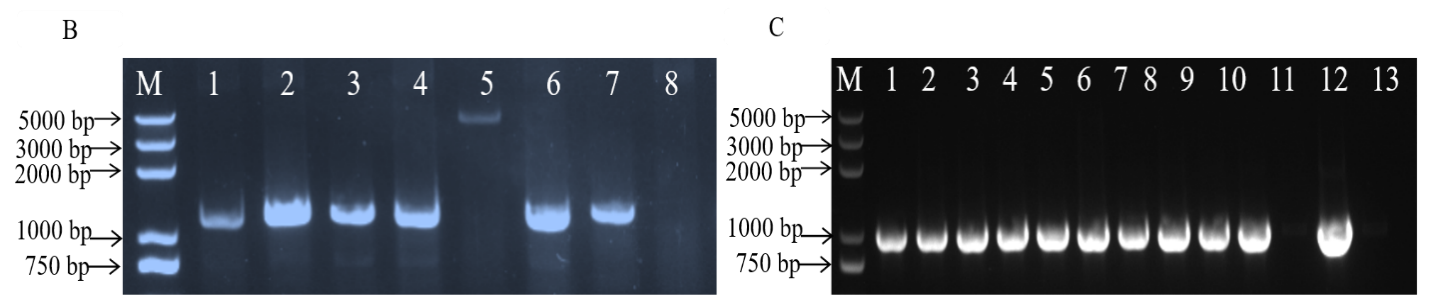


**Figure S4:** **Complement strains using *hsp60* and native promoter (*Np*) confirmation by PCR.** (A) *Np* position in the Mab genome. (B) MabCP*hsp60*-Mab*sigH* confirmed by PCR using primers pMVHsp60-F/pMVrrnB-R. M Lane indicates DNA marker, 1 to 6 transformant colonies, 7 positive (pMV261-*MAB_3543c*), and 8 negative control (Δ*sigH*). The expected length is 1022 bp. (C) CP*Np*Mab*sigH* strain confirmed by PCR using primers pRH3543 F/pMVrrnB-R. M Lane indicates DNA marker, 1 to 11 transformant colonies, 12 positive (pMV261-NP-*MAB_3543c*), and 13 negative controls (Δ*sigH*). The expected length is 857 bp.

**
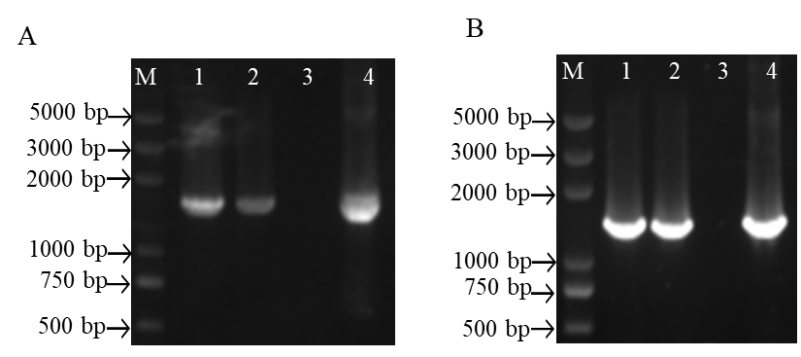
**

**Figure S5: GFP with *Np* or *hsp60* promoter expressing strains confirmation**. Notes: (A) WT*:* pMV-*hsp60*GFP *(*WT: *hsp60-GFP*) and Δ*sigH:* pMV-*hsp60*GFP *(*Δ*sigH*: *hsp60-*GFP) strains confirmation by PCR using primers seqpMV261-F/R. Lane M indicates marker; 1, WT: *hsp60-GFP*; 2, Δ*sigH*: *hsp60-*GFP; 3, Negative control (WT Mab); 4, Positive control (pMV-*hsp60-*GFP). The expected band is 1651 bp. (B) WT: pMV-*Np-*GFP (WT: *Np-*GFP) and Δ*sigH*: pMV-*Np*GFP (Δ*sigH: Np-*GFP) strains. Lane M indicates marker; 1, WT: *Np-*GFP; 2Δ*sigH: Np-*GFP; 3, Negative control (Δ*sigH*); 4, Positive control (pMV-*Np-*GFP). The expected band is 1417 bp.


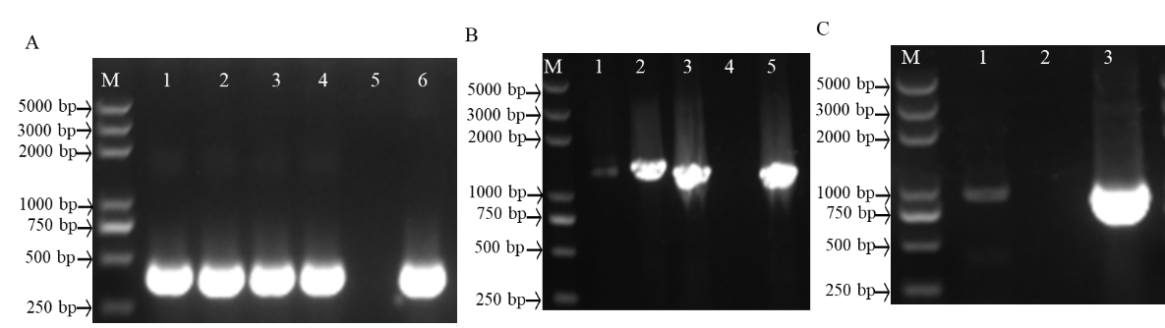


**Figure S6:** **Several overexpressing strains confirmed by PCR using primers** JD-pMV261-F/R. (A) Δ*sigH:* pMV-*MAB_4143c* strains confirmation. Lane M indicates marker; 1 and 4 different colonies; 5, Negative control (Δ*sigH*); 6, Positive control (pMV-*MAB_4143c*). The expected band is 451 bp. (B) Δ*sigH:* pMV-*MAB_3388c* strains confirmation. Lane M indicates marker; 1 and 3 different colonies; 4, Negative control (Δ*sigH*); 5, Positive control (pMV-*MAB_3388c*). The expected band is 1330 bp. (C) Δ*sigH:* pMV-*MAB_1011c* strains confirmation. Lane M indicates marker; 1 colony; 2, Negative control (Δ*sigH*); 5, Positive control (pMV-*MAB_1011c*). The expected band is 952 bp.

A


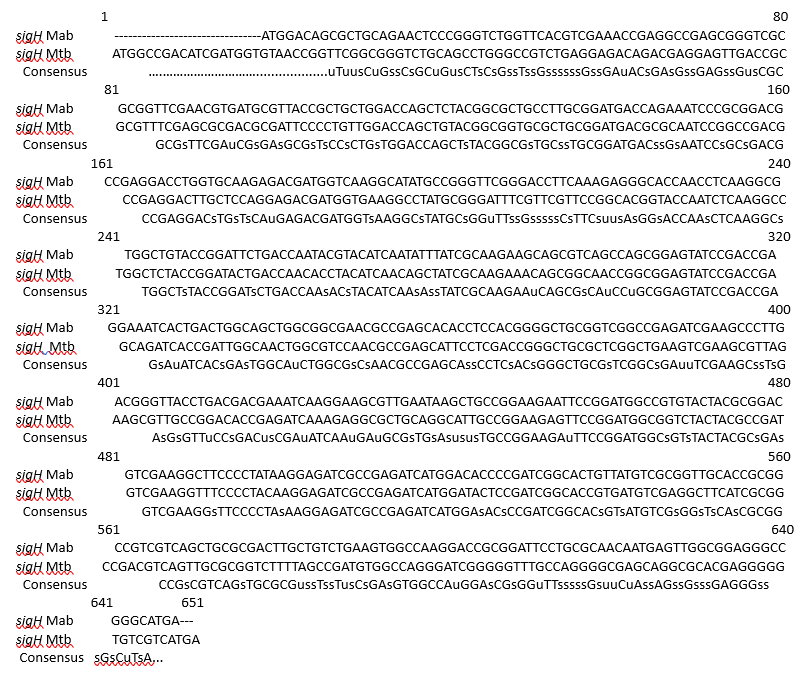


B


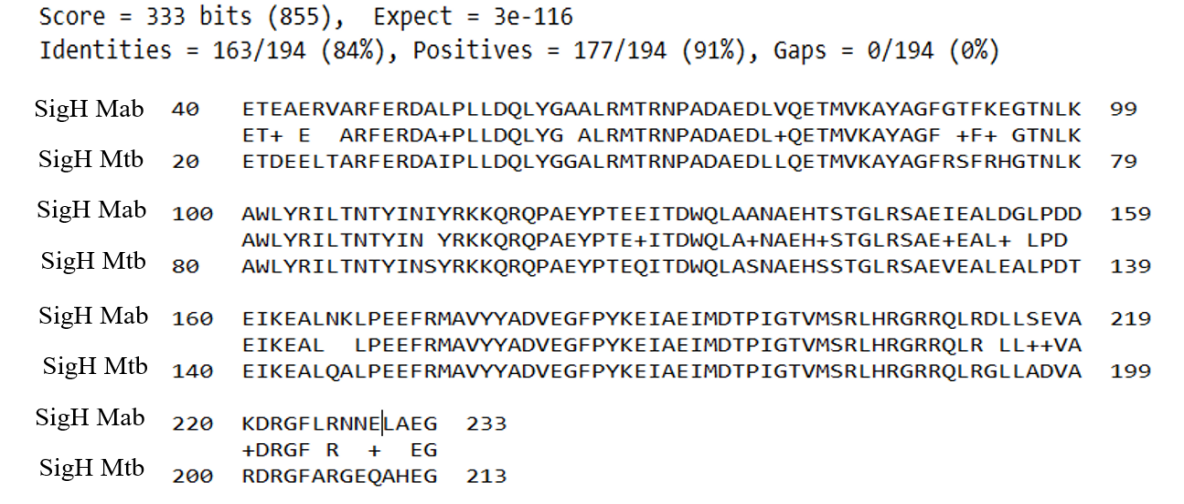


**Figure S7:** **Sequence alignment**. The Nucleotide (A) and protein (B) sequences of *Mycobacterium abscesuss* (Mab) and *Mycobacterium tuberculosis* (Mtb) SigH were compared using Clustal Omega. The two proteins share 84% similarity in their peptide sequences.


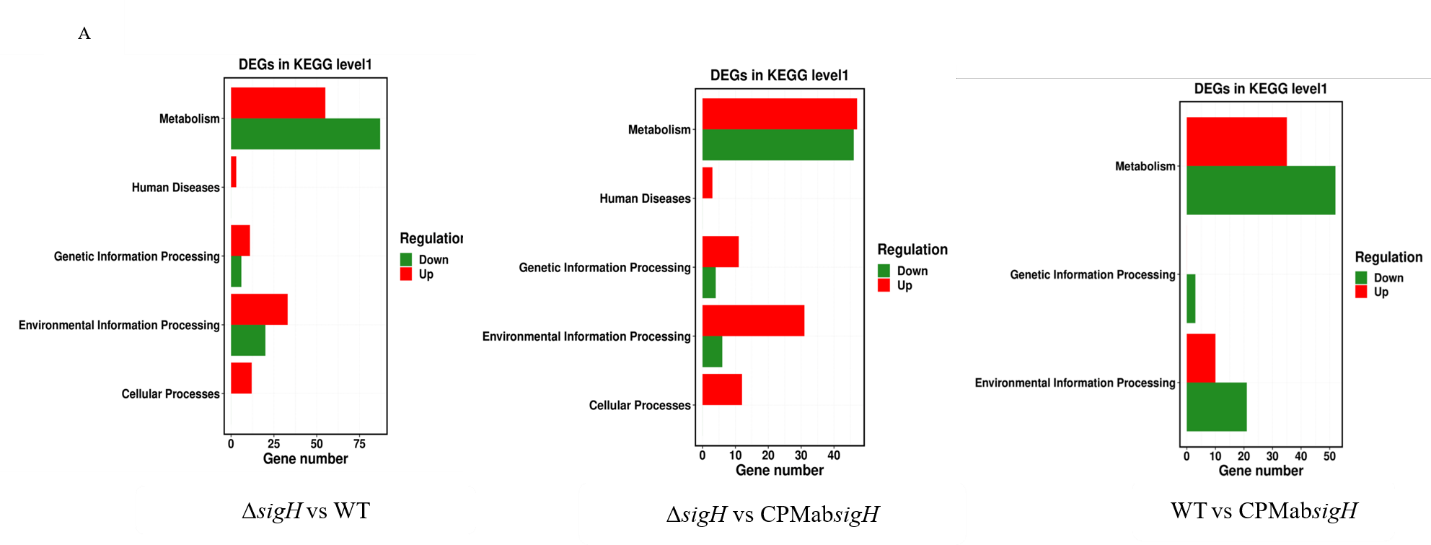


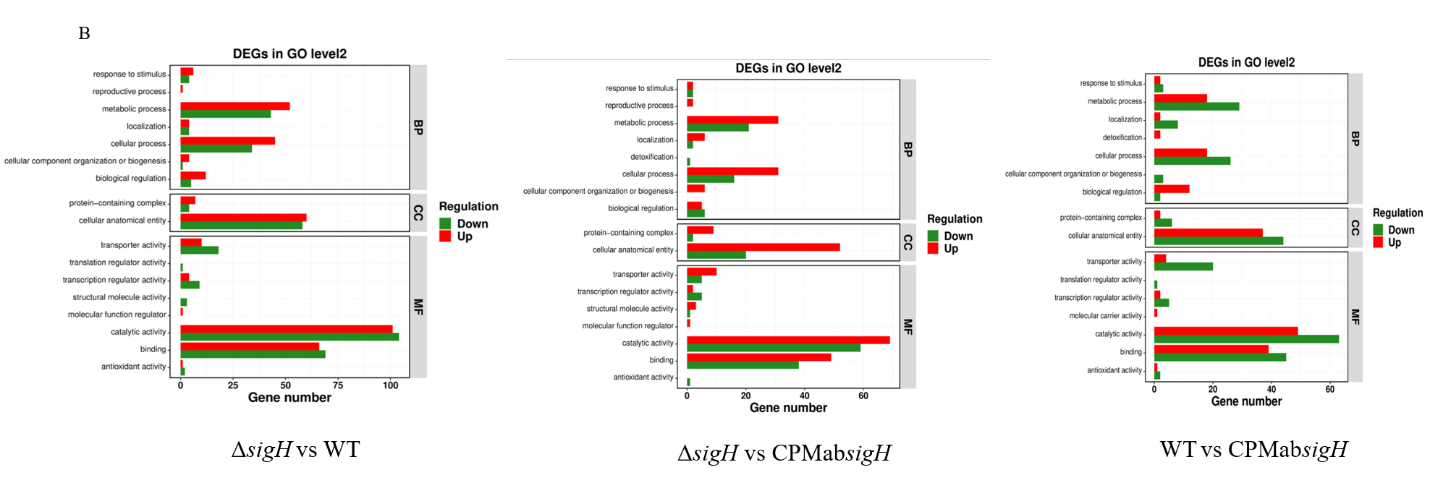


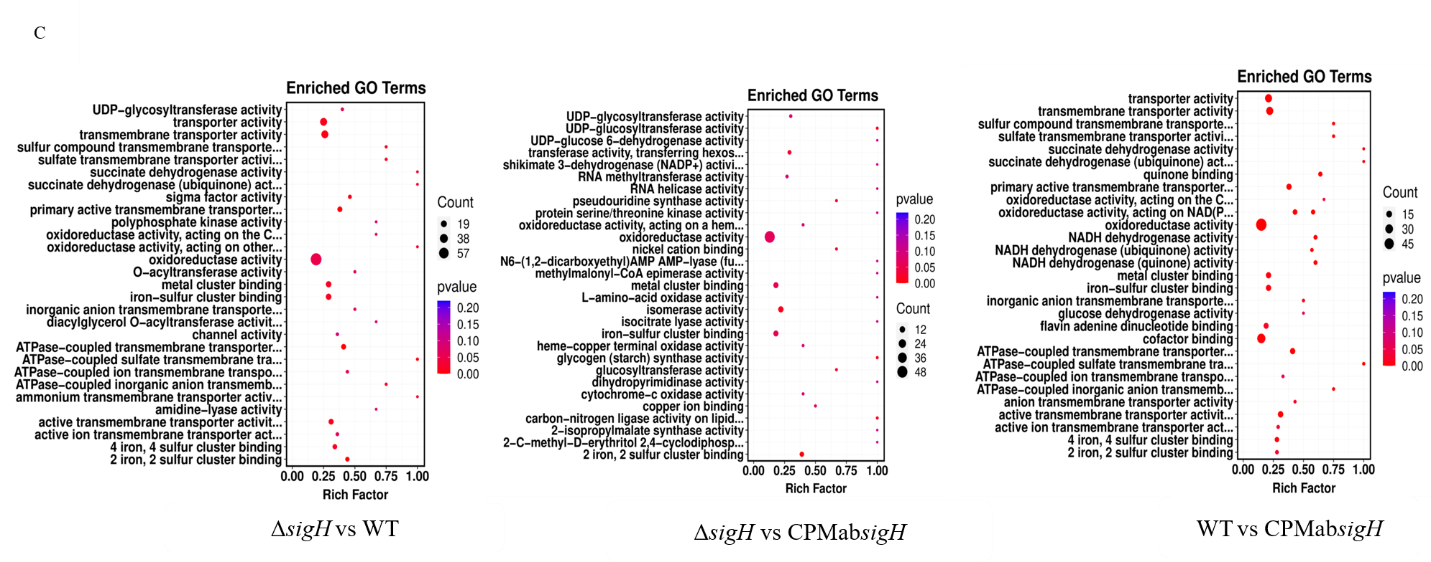


**Figure S8: Functional annotation analysis.** (A) The differentially expressed genes (DEGs) in the comparison group were pathways of KEGG level-1 distribution in the hierarchy. (B) Differentially expressed genes in the comparison group gene ontology (GO) level-2 distribution in the levels. (C) GO functional enrichment analysis based on molecular function (MF).

**Table S1**: List of primer sequences used in his study.

| Primer Names | Nucleotide sequences (5´ – 3´) |
| --- | --- |
| 3543cUp-F | cgctctagaactagtggatccGCTGCACAGAATGCGCTCCC |
| 3543cUp-R | ttgttgcgcaggaatccgGTCACGGCGGTCCTTTCGGTAG |
| 3543cDown-F | acCGGATTCCTGCGCAACAATG |
| 3543cDown-R | ctcgaggtcgacggtatcgatCGCCACCTCGCTCTCGTCAA |
| *sigH*UD-F | cgctctagaactagtggatccGCTGCACAGAATGCGCTCCC |
| *sigH*UD-R | ctcgaggtcgacggtatcgatCGCCACCTCGCTCTCGTCAA |
| cr*sigH*-F | ATCGGCAGCTTATTCAACGCTTCA |
| cr*sigH*-R | AGCTTGAAGCGTTGAATAAGCTGCCGATCT |
| Id3543c-F | TGGACGGCTGGTACGAGTG |
| Id3543c-R | GCCAGGGCGGTTTGGTGAGT |
| pMV-3543c-F or  pMV261- *MAB_3543c* F | ggccaagacaattgcggatccATGGCGTGCCTCGAACGT |
| pMV-3543c-R or  pMV261- *MAB_3543c* R | gttaactacgtcgacatcgaTCATGCCCGGCCCTCCGC |
| pRH3543 F | tcaccatggtggccatctagaGAGAGGCACAGTAACGCCCC |
| pMVHsp60-F | tcaccatggtggccatctagaGGTGACCACAACGACGCG |
| pMVrrnB-R | aacctgcaggtcgactctagaATAAAACGAAAGGCCCAGTCTTT |
| pMV261-sigHmtb F | ggccaagacaattgcggatccAATGGCCGACATCGATGGTG |
| pMV261-sigHmtb R | gttaactacgtcgacatcgatTCATGACGACACCCCCTCG |
| pMV-MAB_1011c F | ggccaagacaattgcggatccGTGACCGCATCAACTTATGTA |
| pMV-MAB_1011c F | gttaactacgtcgacatcgatTTAGCCGCCCAGCCTGGC |
| pMVMAB_4143c F | ggccaagacaattgcggatccATGACGACAGCCCACGGC |
| pMVMAB_4143c R | ggccaagacaattgcggatccTCACCCCTGCGGGTAGAAG |
| pMV-MAB_3388c F | ggccaagacaattgcggatccGTGAATAACCGCGTCACGGT |
| pMV-MAB_3388c R | gttaactacgtcgacatcgatTCAGTCGTCGGGAATGTCGA |
| JD-pMV261-F | CATCCCCGATCCGGAGGAA |
| JD-pMV261-R | CTGGCAGTCGATCGTACGC |
| pNPsigH-GFP F | TACCAGATCTTTAAATCTAGAagctttgacggccttggaa |
| pNPsigH-GFP R | GCCCTTGCTCACCATGGATCCtgtcacctgctataccccttcc |
| seqpMV261-F | TACCAGATCTTTAAATCTAGAagctttgacggccttggaa |
| seqpMV261-R | TACCAGATCTTTAAATCTAGAagctttgacggccttggaa |
| RT sigA F1 | AGCGTGAGCTGCTACAGGAC |
| RT sigA R1 | TGGATTTCCAGCACCTTCTC |
| RT sigA F2 | CACAAAGGGTTACAAGTTCTCG |
| RT sigA R2 | GCTTGTTGATGACCTCGACC |
| RT MAB_3542c F | CTGCGAGGTATCGGGTT |
| RT MAB_3542c R | GCATTCACCGTCGAGGAG |
| RT MAB_3543c F | CTTCAAAGAGGGCACCAACC |
| RT MAB_3543c R | CGCTGCTTCTTGCGATAAA |
| RT MAB_4143c F | GGGTGCTCAATGATGGTGTC |
| RT MAB_4143c R | TGGTCGGACTGGGGAATG |
| RT MAB_2460 F | CCGGATGCGCTGATGGTGGT |
| RT MAB_2460 R | ACTCCATTCGCACAGACTTCG |
| RT MAB_4694 F | GCCGTCACCGTGGAGAAT |
| RT MAB_4694 R | GGAAAGCACATCCCGTCATAG |
| RT MAB_1013 F | TCACGTTTCTGGATTCCCTCA |
| RT MAB_1013 R | CGTTGGACGACGACCTTTT |
| RT MAB_1422c F | TGGACGACGAGAATCCCTTTG |
| RT MAB_1422c R | CGAATCCCGCCTCCTTGA |
| RT MAB_2462 F | CGCCGTATCCGTTGAAGC |
| RT MAB_2462 R | CAACCAATCGCTGGGTGAAG |
| RT MAB_3016 F | CCGCTTGGGTCAGTTGCT |
| RT MAB_3016 R | GGGATGGTGCGCTTCTCA |
| RT MAB_1011c F | TTCGTCGGCATCTACAACAGG |
| RT MAB_1011c R | TGGCCCGGAAGAAGAACG |
| RT MAB_1005c F | CGGTGGACAACCTGTTCGA |
| RT MAB_1005c R | GGTCGCCAGACTTCCCAAT |

**Table S2:** Diamide susceptibility testing against different Mab strains

| Strains | Diamide zone of inhibition in mm *^a^* at concentrations (4 M) *^b^* |
| --- | --- |
| WT | 20 ± 0.707107 |
| Δ*sigH* | 35 ± 1.414214 |
| CPMab*sigH* | 26 ± 2.12132 |

*^a^* Zone of inhibition is determined at mm (Millimeter)

*^b^* Drug concentration used at M (Molar)

**Table S3:** The highest transcriptome changes of DEGs in different groups of Mab for sigma and anti-sigma factor, based on log_2_(fold change) values

| Gene Name | Δ*sigH* vs. WT | | Δ*sigH* vs. CPMab*sigH* | | WT vs. CPMab*sigH* | | Description |
| --- | --- | --- | --- | --- | --- | --- | --- |
|  | Log2F | P value | Log2F | P value | Log2F | P value |  |
| *MAB_3543c* | -12.100632 | 1.6E-100 | -10.675 | 2.476 | -5.51448 | 1.06E-39 | RNA polymerase sigma-E factor |
| *MAB_4143c* | -6.243253 | 0.0000105 | -4.7424 | 5.06E-19 | -1.78279 | 0.013735 | Putative anti-ECF sigma factor ChrR |
| *MAB_1362* | -3.10920 | 1.4E-35 | -2.00985 | 4.12E-17 | -1.09943 | 4.22E-05 | Probable alternative RNA polymerase sigma factor |
| *MAB_4553* | 2.16613 | 1.81E-13 | 0.567474 | 0.018916 | 1.598592 | 1.18E-07 | Probable alternative RNA polymerase sigma factor SigG |
| *MAB_3028* | -1.6659 | 3.67E-15 | -0.8149 | 9.46E-05 | -0.85106 | 4.10E-05 | RNA polymerase sigma factor |
| *MAB_3542c* | 1.1171 | 0.0061704 | -0.96329 | 0.005546 | 2.079557 | 1.61E-07 | Conserved hypothetical protein (possible anti-sigma factor) |
| *MAB_3724c* | -1.0928 | 8.82E-07 | -0.48547 | 0.027627 | -0.60739 | 0.005824 | Probable RNA polymerase sigma-D factor |
| *MAB_2163* | -0.860954 | 0.007539 | -0.61967 | 0.04355 | -0.24157 | 0.487754 | Probable RNA polymerase sigma-70 factor ECF subfamily |
| *MAB_4652* | 0.652824 | 0.782671 | 1.202502 | 0.291049 | -0.53103 | 0.711011 | Putative RNA polymerase sigma factor |
| *MAB_2511* | -0.60470 | 0.032504 | 0.544475 | 0.704911 | -0.434 | 0.119062 | Alternative sigma factor SigF |
| *MAB_3465* | -0.561 | 0.07736 | -1.20197 | 1.89E-05 | 0.640454 | 0.020396 | Putative sulfate transporter/antisigma-factor |
| *MAB_3428* | -0.4695 | 0.04922 | -0.84362 | 0.000412 | 0.374099 | 0.116043 | Probable RNA polymerase sigma-C factor |
| *MAB_4259* | -0.462661 | 0.26039 | 0.565888 | 0.046525 | -0.10327 | 0.7434 | RNA polymerase sigma-70 factor ECF subfamily. |
| *MAB_2513c* | -0.4608 | 0.028615 | -0.67689 | 0.000812 | 0.216047 | 0.269353 | Anti-sigma factor RsbW |
| *MAB_4733* | -0.4349 | 0.356981 | -0.43539 | 0.356981 | 0.000117 | 0.910834 | Putative RNA polymerase sigma factor |
| *MAB_4200* | -0.38966 | 0.319483 | -0.55581 | 0.1155 | 0.165986 | 0.605908 | Putative sigma factor |
| *MAB_3678c* | -0.38541 | 0.058155 | -0.66657 | 0.000737 | 0.281136 | 0.14875 | Probable alternative RNA polymerase sigma factor SigJ |
| *MAB_3009* | -0.3529 | 0.092938 | 0.383199 | 0.06832 | -0.73617 | 0.000489 | Probable RNA polymerase sigma factor RpoD (Sigma-A) |
| *MAB_2512* | 0.3456 | 0.126485 | -0.41265 | 0.062168 | 0.758266 | 0.000879 | Conserved hypothetical protein (sulfate transporter/antisigma-factor antagonist STAS?) |
| *MAB_1248* | -0.30832 | 0.56820 | -0.19004 | 0.727168 | -0.11855 | 0.8109 | Probable RNA polymerase sigma factor |
| *MAB_3888* | -0.29233 | 0.445954 | -0.16294 | 0.71122 | -0.12949 | 0.723856 | Putative RNA polymerase sigma-70 factor |
| *MAB_1126c* | -0.26362 | 0.37537 | 0.365256 | 0.234026 | -0.62851 | 0.046711 | Putative sigma factor |
| *MAB_4938* | -0.18747 | 0.4287 | -0.3084 | 0.208453 | 0.120882 | 0.669209 | Possible alternative RNA polymerase sigma factor SigM |
| *MAB_2511* | -0.6047 | 0.0325 | 0.544475 | 0.704911 | -0.434 | 0.119062 | Alternative sigma factor SigF |

**Table S4:** Predicted promoter elements upstream of *sigH* and selected DEGs.

| No | Gene | Spacer size | -10 | -35 | Satisfy all criteria? | -35 Mismatch |
| --- | --- | --- | --- | --- | --- | --- |
| 1 | *sigH* | 17 bp | aggcacagt | ttgacg | PSIAG | / |
| 2 | *MAB_0219* | 11 bp | ccctagcat | ttgatt | Yes | 2 |
| 3 | *MAB_0686* | 11 bp | tgatctatt | ttgata | PSIAG | 2 |
| 4 | *MAB_0853* | 13 bp | tggtggaat | ctgaat | PSIAG | 3 |
| 5 | *MAB_0885* | 15 bp | tgttcggct | ttaccg | Yes | 2 |
| 6 | *MAB_1012* | 14 bp | cgctaaatt | gtgccc | Yes | 3 |
| 7 | *MAB_1013* | 19 bp | cgccaaaat | ttcctg | Yes | 3 |
| 8 | *MAB_1117c* | 15 bp | gggaatcat | ttgatg | Yes | 1 |
| 9 | *MAB_1362* | 18 bp | ccgcattct | ctgcct | Yes | 3 |
| 10 | *MAB_1422* | 14 bp | gagcatgct | ttcaaa | Yes | 3 |
| 11 | *MAB_1652* | 15 bp | tcctattct | tggccg | PSIAG | 2 |
| 12 | *MAB_1655* | 14 | tcgtcacct | ttgacg | PSIAG | No |
| 13 | *MAB_2216* | 14 bp | tcataggtt | ttcacg | Yes | 1 |
| 14 | *MAB_2459* | 16 bp | ttgtagaag | ttgcaa | PSIAG | 3 |
| 15 | *MAB_2739* | 14 bp | cgctctact | ttcaat | PSIAG | 3 |
| 16 | *MAB_2630* | 14 bp | tgttcgact | ttcacg | Yes | 1 |
| 17 | *MAB_3240* | 13 BP | cggcattgt | ttgatg | PSIAG | 1 |
| 18 | *MAB_3427* | 16 bp | tgtgagact | ttgaca | Yes | 1 |
| 19 | *MAB_3461c* | 18 bp | gggaataat | ttgtcg | PSIAG | 1 |
| 20 | *MAB_3493* | 17 bp | ggccaaact | ttcaca | Yes | 2 |
| 21 | *MAB_3575* | 15 bp | cggtatatg | ttgccg | PSIAG | 1 |
| 22 | *MAB_3655c* | 15 bp | cggtaacgt | ctgacg | Yes | 1 |
| 23 | *MAB_3719c* | 15 bp | atttacaat | gtgtct | Yes | 3 |
| 24 | *MAB_4059* | 19 bp | tgctaaatt | ctgcta | Yes | 4 |
| 25 | *MAB_4122* | 14 bp | ccgcacgat | ttgaca | Yes | 1 |
| 26 | *MAB_4234c* | 15 bp | tggtcaact | tcgccg | PSIAG | 2 |
| 27 | *MAB_4693* | 13 bp | tgccattgt | ttgacc | PSIAG | 1 |
| 28 | *MAB_4694* | 12 bp | cgacaatat | ttgccg | PSIAG | 1 |
| 29 | *MAB_4695* | 13 bp | tcgtactct | ctgaga | Yes | 3 |
| 30 | *MAB_4699* | 15 bp | acgcatgat | atgtca | Yes | 3 |

***Notes****:* Promoter sequences in adjacent gene = PSIAG

**Table S5:** Predicted promoter elements upstream of sigH and randomly selected genes as a negative control.

| No | Gene | spacer size | -10 | -35 | Satisfy all criteria? | -35 Mismatch |
| --- | --- | --- | --- | --- | --- | --- |
| 1 | *sigH* | 17 bp | aggcacagt | ttgacg | PSIAG | / |
| 2 | *MAB_0006* | 11 bp | cagtaggat | gtgccg | Yes | 3 |
| 3 | *MAB_0471* | 19 bp | ggccacaat | ttcgca | Yes | 3 |
| 4 | *MAB_0480* | 11 bp | ctcaaagat | atgtca | PSIAG | 3 |
| 5 | *MAB_0533* | 18 bp | gggtatacc | ctcaag | PSIAG | 4 |
| 6 | *MAB_3400* | 15 bp | atatatggt | ttaact | Yes | 2 |
| 7 | *MAB_3618* | 18 bp | cggtcaaat | tagtcg | Yes | 2 |
| 8 | *MAB_0805* | 21 bp | cggtatacg | ttgtca | Yes | 2 |
| 9 | *MAB_4350c* | 13 bp | gggcatatt | atgact | Yes | 2 |
| 10 | *MAB_1059c* | 14 bp | cgttctaat | tcgaca | Yes | 2 |

***Notes****:* Promoter sequences in adjacent gene = PSIAG
